# The genetic regulatory signature of type 2 diabetes in human skeletal muscle

**DOI:** 10.1038/ncomms11764

**Published:** 2016-06-29

**Authors:** Laura J. Scott, Michael R. Erdos, Jeroen R. Huyghe, Ryan P. Welch, Andrew T. Beck, Brooke N. Wolford, Peter S. Chines, John P. Didion, Narisu Narisu, Heather M. Stringham, D. Leland Taylor, Anne U. Jackson, Swarooparani Vadlamudi, Lori L. Bonnycastle, Leena Kinnunen, Jouko Saramies, Jouko Sundvall, Ricardo D'Oliveira Albanus, Anna Kiseleva, John Hensley, Gregory E. Crawford, Hui Jiang, Xiaoquan Wen, Richard M. Watanabe, Timo A. Lakka, Karen L. Mohlke, Markku Laakso, Jaakko Tuomilehto, Heikki A. Koistinen, Michael Boehnke, Francis S. Collins, Stephen C. J. Parker

**Affiliations:** 1 Department of Biostatistics and Center for Statistical Genetics, University of Michigan, Ann Arbor, Michigan 48109, USA; 2 National Human Genome Research Institute, National Institutes of Health, Bethesda, Maryland 20892, USA; 3 European Bioinformatics Institute, European Molecular Biology Laboratory, Wellcome Trust Genome Campus, Hinxton, Cambridgeshire CB10 1SD, UK; 4 Department of Genetics, University of North Carolina, Chapel Hill, North Carolina 27599, USA; 5 Department of Health, National Institute for Health and Welfare, P.O. Box 30, Helsinki FI-00271, Finland; 6 South Karelia Central Hospital, Lappeenranta 53130, Finland; 7 Department of Computational Medicine & Bioinformatics, University of Michigan, Ann Arbor, Michigan 48109, USA; 8 Center for Genomic & Computational Biology, Duke University, Durham, North Carolina 27708, USA; 9 Department of Pediatrics, Division of Medical Genetics, Duke University Medical Center, Durham, North Carolina 27708, USA; 10 Department of Preventive Medicine, Keck School of Medicine of USC, Los Angeles, California 90089, USA; 11 Department of Physiology and Biophysics, Keck School of Medicine of USC, Los Angeles, California 90089, USA; 12 Institute of Biomedicine/Physiology, University of Eastern Finland, Kuopio FI-00100, Finland; 13 Kuopio Research Institute of Exercise Medicine, Kuopio FI-00100, Finland; 14 Department of Clinical Physiology and Nuclear Medicine, Kuopio University Hospital, University of Eastern Finland, Kuopio FI-00100, Finland; 15 Department of Medicine, University of Eastern Finland, Kuopio FI-00100, Finland; 16 Kuopio University Hospital, Kuopio FI-00100, Finland; 17 Chronic Disease Prevention Unit, National Institute for Health and Welfare, P.O. Box 30, Helsinki FI-00271, Finland; 18 Center for Vascular Prevention, Danube University Krems, Krems 3500, Austria; 19 Diabetes Research Group, King Abdulaziz University, Jeddah 21589, Saudi Arabia; 20 Dasman Diabetes Institute, Dasman 15461, Kuwait; 21 Department of Medicine and Abdominal Center: Endocrinology, University of Helsinki and Helsinki University Central Hospital, P.O. Box 340, Haartmaninkatu 4, Helsinki FI-00029, Finland; 22 Minerva Foundation Institute for Medical Research, Biomedicum 2U, Tukholmankatu 8, Helsinki FI-00290, Finland; 23 Department of Human Genetics, University of Michigan, Ann Arbor, Michigan 48109, USA

## Abstract

Type 2 diabetes (T2D) results from the combined effects of genetic and environmental factors on multiple tissues over time. Of the >100 variants associated with T2D and related traits in genome-wide association studies (GWAS), >90% occur in non-coding regions, suggesting a strong regulatory component to T2D risk. Here to understand how T2D status, metabolic traits and genetic variation influence gene expression, we analyse skeletal muscle biopsies from 271 well-phenotyped Finnish participants with glucose tolerance ranging from normal to newly diagnosed T2D. We perform high-depth strand-specific mRNA-sequencing and dense genotyping. Computational integration of these data with epigenome data, including ATAC-seq on skeletal muscle, and transcriptome data across diverse tissues reveals that the tissue-specific genetic regulatory architecture of skeletal muscle is highly enriched in muscle stretch/super enhancers, including some that overlap T2D GWAS variants. In one such example, T2D risk alleles residing in a muscle stretch/super enhancer are linked to increased expression and alternative splicing of muscle-specific isoforms of *ANK1*.

## Abstract (web summary)

More than 90% of genetic variants associated with type 2 diabetes occur in non-coding regions. Scott *et al*. report genomes, epigenomes and transcriptomes of skeletal muscle from 271 participants with a range of glucose tolerances, revealing a genetic regulatory architecture enriched in muscle stretch/super enhancers.

## Graphical abstract

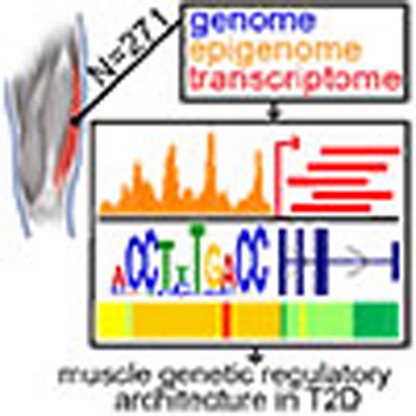

The prevalence of diabetes in 2015 reached 415 million adults worldwide, and is projected to increase to 642 million by 2040 (www.idf.org/diabetesatlas). T2D accounts for ∼90% of these individuals^1^. Pancreatic islet beta-cell dysfunction, accentuated by insulin resistance in skeletal muscle and other peripheral tissues, are the hallmarks of T2D^2^. To examine the relationship between T2D and related traits and muscle metabolism, we obtained blood samples and skeletal muscle biopsies, and performed clinical phenotyping, including oral glucose tolerance tests (OGTT), on 271 Finnish individuals. Study subjects were chosen to span the range of glucose tolerance from normal to newly diagnosed (not on glucose-lowering drug therapy) T2D.

Because most (>90%) T2D and related trait GWAS single-nucleotide polymorphisms (SNPs) reside in non-coding regions, we aim to refine the molecular mechanisms underlying these associations by identifying the target gene(s) and direction of effect the risk allele has on target gene expression. To accomplish this, we further perform dense and diverse molecular profiling on this collection of skeletal muscle biopsies at the genome, epigenome and transcriptome level. Integration of these data types allowed us to nominate multiple T2D GWAS SNP effector transcripts. These genetic, epigenomic and transcriptomic results represent the largest study of the regulatory landscape in human muscle and reveal how it relates to T2D.

## Results

### T2D insights from mRNA-seq maps and clinical phenotypes

**Figure 1:**
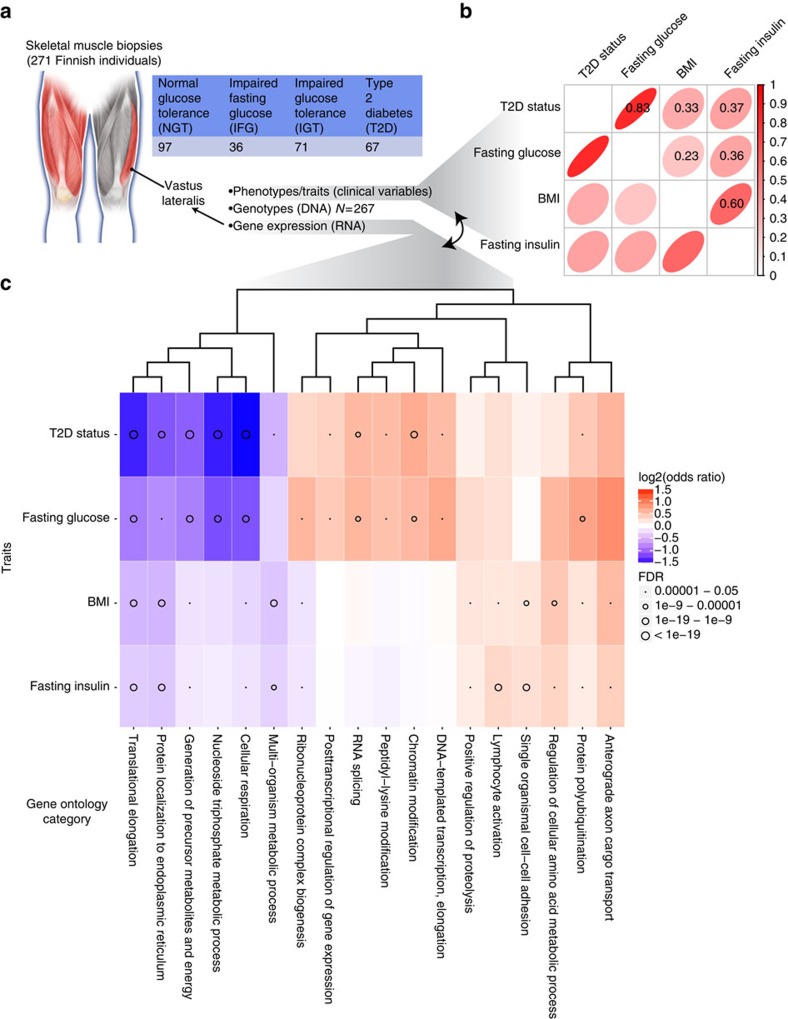
Molecular profiling maps of skeletal muscle combined with dense phenotyping reveals insights about T2D. (**a**) To understand the full spectrum of genetic variation and regulatory element usage in T2D-relevant tissue and across disease progression, we obtained skeletal muscle biopsies from the vastus lateralis of 271 well-phenotyped Finnish individuals with normal and impaired glucose tolerance, impaired fasting glucose or newly diagnosed, untreated T2D. (**b**) Correlogram of Spearman rank correlation coefficients for key metabolic traits. (**c**) Heatmap of GO terms for differentially expressed genes. For each trait, the 20 GO terms most significantly enriched for positive expression–trait association and the 20 GO terms most significantly enriched for negative expression–trait association were selected. GO terms were pruned to eliminate redundant terms. The terms were hierarchically clustered using the GO term enrichment beta. Darker red, stronger positive gene expression–trait association; darker blue, stronger negative association. Circle size represents number of significant GO terms.

We sequenced the mRNA from study participants (Fig. 1a, Supplementary Table 1) to a mean depth of 91.3M strand-specific paired-end reads, the most comprehensive human skeletal muscle transcriptome catalogue to date^3^,^4^,^5^. We tested for (1) differential gene expression between individuals with T2D and normal glucose tolerance (NGT) and (2) association between gene expression and three T2D-related traits (fasting glucose, fasting insulin and body mass index (BMI)) (Fig. 1b), adjusting for covariates (see Methods). At a false discovery rate (FDR) of 5%, we found three genes differentially expressed with T2D status; most strongly differentially expressed was a positive regulator of senescence, ubinuclein 1 (*UBN1*) (*β*=0.83, *q*-value=0.0089). At FDR 5%, we detected a wide range in the number of gene–trait associations, from 38 for fasting glucose to 6,080 for fasting insulin.

To probe the underlying biology of differential gene expression, we performed gene ontology (GO) term analysis for each trait using the strength and direction of trait–gene association (−log_10_(*P* value) signed for the direction of association) as a predictor of GO term membership^6^. We display a pruned list of the most strongly associated GO terms, selected separately for terms enriched for genes with positive and negative trait association (Fig. 1c, Supplementary Fig. 1).

With T2D status, and with increases in fasting glucose, fasting insulin and BMI, we observed lower expression of genes involved in endoplasmic reticulum protein localization and translational elongation. For T2D, the most significant trends were for decreased expression of cellular respiration genes (*q*-value=1.4 × 10^−35^), consistent with previous observations in skeletal muscle samples from T2D and NGT individuals following hyperinsulinemic–euglycemic clamp^7^. Mitochondrial regulatory protein PGC-1alpha (PPARGC1A) was identified by Mootha *et al*.^7^ as a potential master regulator of mitochondrial expression. We observed lower, non-significantly different expression levels of *PPARGC1A* (*β*=−0.24, *q*-value=0.57) in individuals with T2D. Decreased mitochondrial function is a component of the mTOR pathway which is dysregulated in metabolic diseases; downregulation of the pathway shifts cells away from protein synthesis and cell growth and towards protein catabolism^8^. Consistent with this, for T2D, we observed lower expression of genes involved in generation of precursor metabolites, translational elongation and higher expression of genes involved in protein polyubiquitination (Fig. 1c).

Interestingly, higher expression levels of genes for leucocyte activation were strongly associated with higher levels of fasting insulin (*q*-value=1.3 × 10^−14^) and less strongly with other traits. This could suggest that muscle samples from individuals with insulin resistance have increased inflammatory cell infiltration. However, this association remained when we estimated and then adjusted for the fraction of white blood cells (WBCs) and lymphocytes in the muscle samples (see Methods) in the trait–gene expression analysis (*q*-value=2.5 × 10^−14^, Supplementary Fig. 2).

### Genetic and epigenomic regulatory architecture of muscle

To understand how genetic variation may influence muscle gene expression and thus T2D and related traits, we successfully genotyped 267 of the 271 samples using the Illumina Omni2.5 array and imputed 8,406,237 SNPs with minor allele frequency >0.01 using the Genetics of Type 2 Diabetes (GoT2D) reference panel (Methods). We tested for association of variants within 1 Mb of the transcription start site (TSS) with gene expression (cis-eQTL). At FDR=5%, we detected 2,104,118 cis-eQTLs in 19,697 (92%) of 21,420 tested genes (Supplementary Table 2), of which 168,633 cis-eQTLs remained when pruned on a per-gene basis by linkage disequilibrium (LD) at *r*^2^<0.2. cis-eQTLs cluster at the TSS (Supplementary Fig. 3), as previously noted^9^,^10^. Within 15,449 tested protein-coding genes, 14,479 (93.6%) had ⩾1 cis-eQTLs. Using a complementary approach, we tested for allele-specific expression (ASE) in protein-coding genes that had a heterozygous-transcribed SNP in ⩾10 individuals; we detected ASE at FDR=5% in 7,404 (80.2%) of 9,228 tested genes (see Supplementary Fig. 4 for overlap with cis-eQTL). We compared the total set of our cis-eQTL results to those of 361 genotype-tissue expression (GTEx)^5^ skeletal muscle samples for 95.5M SNP–gene pairs tested in both studies (Supplementary Fig. 5). Of these SNP–gene pairs, in our muscle sample at FDR=5% we detected 3.5M SNP–gene pairs, of which 83.8% had a concordant direction in GTEx. Comparing our catalogue to the 1.1M GTEx SNP–gene pairs (FDR=5%), 813K SNP–gene pairs were significantly associated in both studies; 99% of these had concordant effect directions.

**Figure 2:**
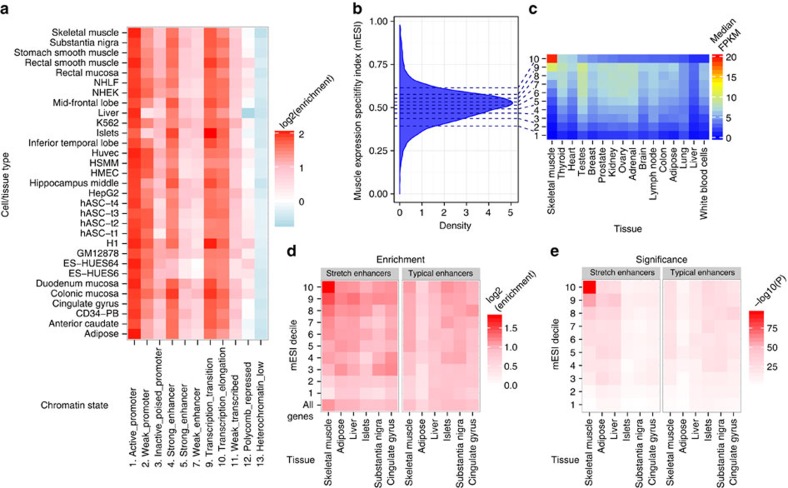
The genetic regulatory architecture of muscle-specific gene expression. (**a**) Muscle cis-eQTL enrichment across chromatin states in diverse cell or tissue types^11^,^15^,^63^,^64^. (**b**) Genes that are both highly expressed in skeletal muscle and highly tissue-specific fall into muscle expression specificity index (mESI) decile 10; genes which are lowly and ubiquitously expressed fall in decile 1. (**c**) As mESI decile increases, genes have greater expression in skeletal muscle but not in other tissue types. (**d**,**e**) cis-eQTL genes stratified by mESI decile. cis-eQTLs which fall into muscle stretch enhancers (⩾3 kb) are significantly enriched for genes expressed highly and specifically in muscle versus cis-eQTLs in typical enhancers (≤800 bp).

Previous studies of blood and lymphoblastoid cell lines have shown enrichment of cis-eQTLs that overlap enhancer and promoter regions^9^,^10^. To explore this relationship in muscle, we created chromatin state maps using ChromHMM^11^ for skeletal muscle and 30 diverse cell or tissue types, including adipose, liver and pancreatic islets (Supplementary Fig. 6; see Methods). To assess the relationship between our skeletal muscle cis-eQTLs and chromatin states, we calculated enrichment statistics for how these different features overlap while controlling for minor allele frequency, distance to TSS and the number of SNPs in LD^12^. We observed enrichment of skeletal muscle cis-eQTLs in genomic regions with active (promoter, enhancer or transcribed) chromatin states and a depletion in those with inactive (low signal) chromatin states across a broad range of cell types (Fig. 2a). This general enrichment pattern may reflect the predominance of cis-eQTLs from widely expressed housekeeping genes and mask a smaller set of muscle-specific genes.

To develop a measure of muscle gene expression specificity, we analysed additional RNA-seq data representing a set of diverse tissues from the Illumina Human Body Map 2.0 project. We used an information theory approach^13^,^14^ to score genes based on muscle expression level and specificity relative to the panel of 16 diverse tissues; we refer to this score as the muscle expression specificity index (mESI; Methods) (Fig. 2b). We divided the genes into mESI deciles, where the 1st decile represents genes that are expressed at uniformly low levels across all tissues and the 10th decile represents genes that are highly and specifically expressed in muscle (Fig. 2c). cis-eQTL SNPs for genes in the 10 deciles were collected and partitioned into corresponding bins, which showed a larger range in skeletal muscle chromatin state enrichments (Supplementary Fig. 7) compared with the general non-mESI-based enrichments (Fig. 2a).

We previously defined chromatin ‘stretch enhancers' as long (⩾3 kb) regions of tissue-specific active chromatin, and found they are highly enriched for GWAS variants in disease-relevant tissues^15^,^16^. Here we observed increasing cis-eQTL enrichment with increasing mESI decile in skeletal muscle stretch enhancers, but neither in muscle ‘typical enhancers' (≤800 bp; median enhancer size) nor in stretch and typical enhancers from other tissues (Fig. 2d, Supplementary Fig. 8). Enrichment remained at different length thresholds (longest 10, 5, 1%) for calling stretch enhancers (Supplementary Fig. 9). Super enhancers^17^,^18^, defined based on the abundance of acetylated lysine 27 on histone H3 (ref. 19), substantially overlap stretch enhancers (Supplementary Fig. 10) and show similar muscle cis-eQTL enrichment trends (Supplementary Fig. 11). These results suggest that the genetic regulatory architecture of muscle-specific gene expression is preferentially encoded in muscle stretch/super enhancers relative to typical enhancers.

**Figure 3:**
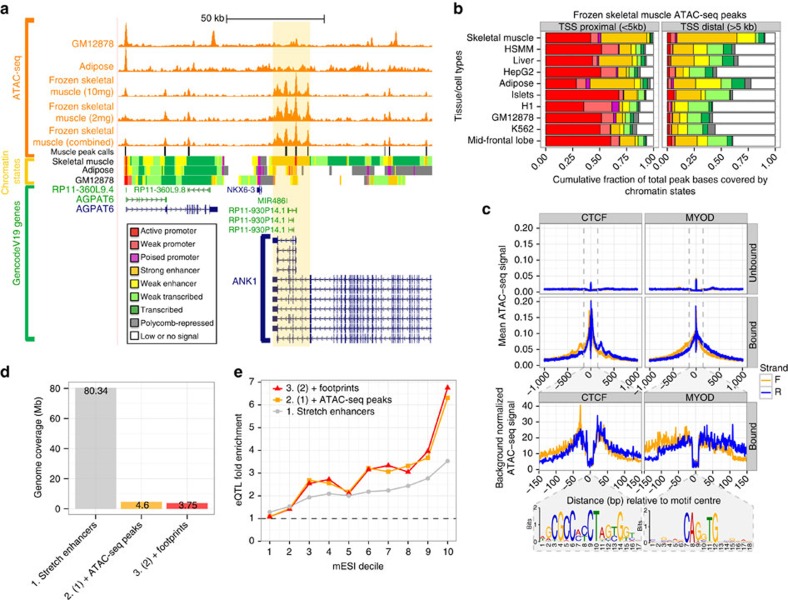
ATAC-seq maps in frozen skeletal muscle. (**a**) ATAC-seq profiles in skeletal muscle replicates and combined compared with similar profiles in adipose^21^ and the lymphoblastoid cell line GM12878 (ref. 20), and to reference chromatin state maps in diverse cell or tissue types. (**b**) Skeletal muscle combined replicate ATAC-seq peak calls show enrichment for skeletal muscle-active chromatin states, which is pronounced at TSS-distal regions. (**c**) Example ATAC-seq footprint aggregate plots at CTCF and MYOD sites. (**d**) Relative genome coverage by different classes of skeletal muscle regulatory elements. (**e**) Skeletal muscle cis-eQTL enrichments at different mESI bins relative to the regulatory elements described in **d**.

Regulatory information encoded in DNA is activated through the binding of transcription factors (TFs) that can alter nucleosomal architecture and increase local chromatin accessibility. Because the stretch and super enhancer maps are based on modified histone data sets, it is difficult to detect these local TF-binding events. To construct higher-resolution regulatory maps across skeletal muscle and specifically within the larger stretch enhancer regions, we performed open chromatin accessibility mapping in frozen skeletal muscle using ATAC-seq^20^. We found that ATAC-seq performed on frozen skeletal muscle is reproducible using 10 and 2 mg tissue inputs (Fig. 3a, Supplementary Fig. 12), and therefore we combined these replicates for further analyses. Comparison of our skeletal muscle ATAC-seq maps with similar maps from adipose^21^, the lymphoblastoid cell line GM12878 (ref. 20), and with our chromatin states at the *ANK1* locus revealed striking muscle-specific chromatin architecture that is consistent with the chromatin state maps (Fig. 3a, orange-highlighted region). For example, the skeletal muscle ATAC-seq peak calls occur preferentially at skeletal muscle promoter and enhancer chromatin states. Applying this analysis genome wide, we found a high degree of correspondence between the peak calls and active chromatin states (Fig. 3b). When considering only TSS-distal (>5 kb away from a TSS) ATAC-seq peaks, the overlap with skeletal muscle strong enhancer chromatin states is the highest across all tissues (Fig. 3b, Supplementary Fig. 13).

To obtain an even higher-resolution regulatory map, we performed TF-binding site (TFBS) footprinting analyses, using the CENTIPEDE algorithm^22^. This analysis predicts TF binding based on the occurrence of a motif and the pattern of ATAC-seq transposition events surrounding it. To detect motif occurrences that could be altered by the presence of alleles not in the reference genome, we used a SNP-aware motif scanning approach (see Methods). We detected high-quality footprints for the ubiquitous transcriptional insulator CCCTC-binding factor (CTCF) and the tissue-specific regulator MYOD (Fig. 3c), in addition to many other factors (see Methods). Notably, at nucleosome-size distances adjacent to the CTCF footprint regions we observe phased spikes in the ATAC-seq signal (Fig. 3c, left column middle row), consistent with the known nucleosome-phasing properties of CTCF^23^.

The collection of ATAC-seq peaks and TFBS footprints define progressively smaller regions within muscle stretch enhancers (Fig. 3d), and these regions are progressively more enriched to overlap cis-eQTL at increasing mESI deciles (Fig. 3e). Collectively, these results demonstrate the high quality of our frozen skeletal muscle ATAC-seq data and help to refine the location of transcriptional regulatory variation, suggesting that such maps can be used to identify potentially causal TFBSs that drive cis-eQTL signals.

### Linking GWAS SNPs to effector transcripts in muscle

We and others previously demonstrated that stretch/super enhancers in disease-relevant tissues are highly enriched for GWAS-disease-associated SNPs^15^,^19^, and a recent study identified autoimmune GWAS SNPs that reside in a T-cell super enhancer and act as cis-eQTL^24^. However, no T2D GWAS cis-eQTLs in stretch/super enhancers have been identified in any tissues. To identify genetic regulatory signatures that may contribute to the diabetes phenotype, we assessed the overlap of our muscle cis-eQTL catalogue with 225 GWAS SNPs associated with T2D and 7n T2D-related traits (see Methods). Of the 220 GWAS SNPs assessed in our study, 99 SNPs in 218 GWAS SNP–gene pairs (of a total 4,545 GWAS SNP–gene pairs) had ⩾1 significantly associated genes. We performed iterative conditional analysis to identify GWAS cis-eQTL SNPs likely to be independent of SNPs with substantially stronger expression associations in the same gene (see Methods). 53 variants in 78 GWAS SNP–gene pairs (59 unique genes) remained associated (FDR <5% for the conditional analysis); of these 38 of the 53 variants remained after pruning at *r*^2^<0.2. The top conditional cis-eQTL signals for GWAS SNPs are shown in Table 1; the full list is shown in Supplementary Data 1.

**Figure 4:**
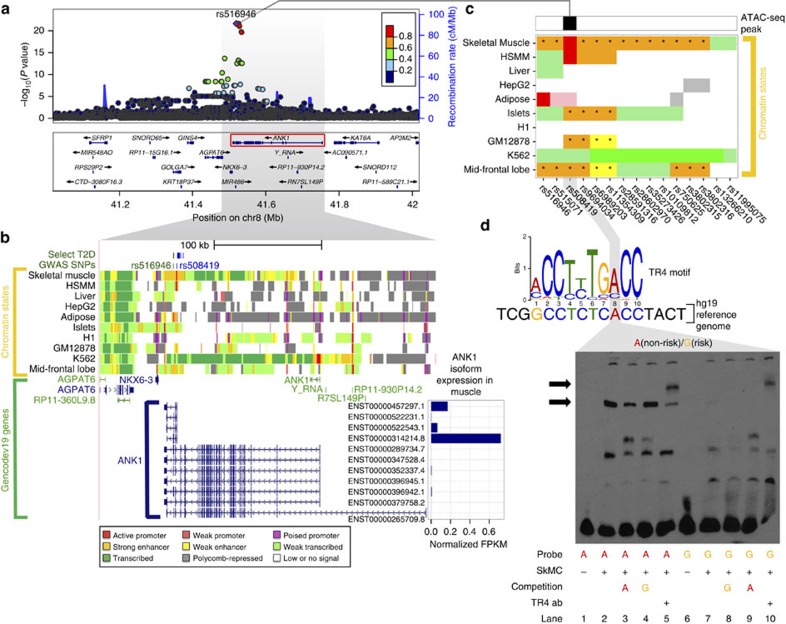
T2D GWAS SNPs in a muscle-specific stretch enhancer of *ANK1* provide mechanistic insights into T2D pathophysiology in skeletal muscle. (**a**) Regional association plot showing the significance of SNPs (points) associated with expression of *ANK1* (highlighted with red rectangle), where the best cis-eQTL rs516946 (purple point) is a T2D GWAS SNP. (**b**) UCSC genome browser view of chromatin states near *ANK1* isoforms. The chromatin states between skeletal muscle and other T2D relevant cell types (adipose, liver, islets) are markedly different. *ANK1* is associated with hereditary spherocytosis, a disease of the red blood cells, which is consistent with the transcribed chromatin states in K562, a myelogeneous leukaemia line of the erythroleukemia type. T2D and related trait GWAS SNPs (dark green) and SNPs in strong LD (*r*^2^⩾0.8; blue) are found within muscle-specific stretch enhancers. *ANK1* expression is shown for each isoform and normalized so that the sum over all isoforms is 1. (**c**) The chromatin states across T2D relevant tissues or cells for SNPs in strong LD (*r*^2^⩾0.8) with T2D GWAS SNP rs516946. Location of the ATAC-seq peak is noted. Asterisks denote SNPs that reside in stretch (⩾3 kb) enhancers. Colour-coding of chromatin is as shown in **b**. (**d**) EMSA using human skeletal muscle cell (SkMC) nuclear extract demonstrates allele-specific binding (see lower horizontal arrow on the left side of the gel) for the non-risk allele (A) of rs508419. A supershift (see upper horizontal arrow on the left side of the gel) using the TR4 antibody shows that TR4 participates in the allele-specific binding.

We observed a top T2D GWAS SNP cis-eQTL signal for the muscle-specific (mESI decile=10) gene *ANK1* (Fig. 4a), where the T2D risk allele at rs516946 resulted in increased gene expression (Table 1). Although the underlying molecular mechanisms were unknown at the time, this locus was first reported as being associated with T2D^25^,^26^, the results we present here help define the impact on skeletal muscle gene expression. This cis-eQTL SNP resides in a skeletal muscle stretch/super enhancer (Fig. 4b,c, Supplementary Fig. 14). Notably, there are no amino-acid-altering variants in strong LD (*r*^2^⩾0.8) with rs516946, but several overlap active chromatin marks in skeletal muscle tissue and human skeletal muscle myoblast (HSMM) cells (Supplementary Fig. 15). One such SNP, rs508419, is flanked by skeletal muscle stretch enhancers, resides in an active promoter specific to skeletal muscle and HSMM, and overlaps an ATAC-seq peak in our muscle data (Fig. 4c). This active promoter ATAC-seq peak SNP is immediately upstream of the TSS for highly expressed short isoforms of *ANK1* (Fig. 4b, see inset bar plot) and is predicted by our SNP-aware motif scans to disrupt a TR4-binding site, consistent with what is reported in the HaploReg database^27^.

We tested each allele of rs508419 in an electrophoretic mobility shift assay (EMSA) using nuclear extract from human skeletal muscle cells (SkMC). We found that the non-risk allele is preferentially bound compared with the risk allele (Fig. 4d). This allelic effect was replicated in both human (HSMM) and mouse (C2C12) myoblast cells (Supplementary Fig. 16). Our ATAC-seq footprinting results indicate that TR4 is bound at this position (CENTIPEDE posterior probability=1), and we observe an allele-specific supershift in the EMSA using a TR4 antibody (Fig. 4d). Together, these results suggest that TR4 binding at the non-risk allele is linked to transcriptional repression, consistent with previous reports about TR4 activity^28^,^29^, and that TR4 binding and repression is disrupted by the T2D risk allele at rs508419, which results in increased *ANK1* expression.

**Figure 5:**
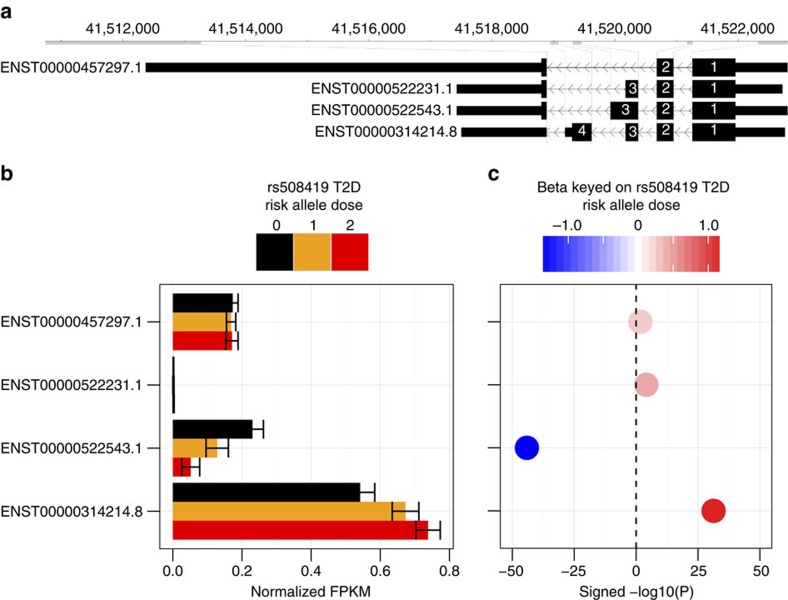
Truncated *ANK1* isoforms are expressed in muscle and have differential splicing associated with rs508419 genotype. (**a**) The four short isoforms of *ANK1*. (**b**) The mean expression of each isoform normalized by the total *ANK1* expression is stratified by genotype; error bars represent ±1 s.d. *N*=8/82/174 for risk allele dosages 0/1/2. (**c**) The direction of effect (*β*) for splicing associated with the rs508419 risk allele (*y*-axis labels are the same as in **b**).

Further splicing (sQTL) and exon usage (exonQTL) investigation (see Methods) of the muscle mRNA-seq data revealed complex splicing patterns that are not apparent when considering bulk gene expression. sQTL analysis of the four short *ANK1* isoforms with a TSS near the active muscle promoter rs508419 SNP (Fig. 5a) showed that abundance for two of the isoforms (ENST00000522543.1 and ENST00000314214.8) is significantly affected by rs508419 genotype, whereas the other two (ENST00000457297.1 and ENST00000522231.1) appear to be invariant (Fig. 5b,c). Interestingly, the two variable transcripts change expression by rs508419 T2D risk allele copies in opposite directions: ENST00000522543.1 decreases expression while ENST00000314214.8 increases it (Fig. 5c). We confirmed this sQTL analysis using an independent exonQTL analysis, quantitative reverse transcription–PCR and droplet digital PCR (ddPCR) (Supplementary Figs 17–20).

## Discussion

Protein products of the small *ANK1* isoforms we identified as linked to a T2D GWAS SNP through our skeletal muscle cis-eQTL map interact with obscurin^30^,^31^, a critical component of, and required for, proper sarcoplasmic reticulum (SR) assembly^32^,^33^. The SR is involved in insulin action on glucose uptake through the regulation of GLUT4 translocation to the plasma membrane (reviewed in ref. 34). Recently, ANK1 was shown to interact with IRS1 in skeletal muscle^35^. IRS1 is indispensable in insulin action on glucose uptake in human SkMCs^36^. Thus, ANK1 is linked to glucose uptake in muscle, and alterations to its expression might perturb this process leading to an insulin-resistant state. However, how the different small *ANK1* isoforms influence insulin-stimulated glucose uptake is presently unclear. Functional studies to better characterize this process are needed.

In summary, we report here on a genome-wide genetic and mRNA-seq analysis of the largest set of clinically characterized human muscle samples described to date. We observed lower expression in T2D individuals for genes involved with cellular respiration, consistent with a previous smaller study^7^. We demonstrated that the genetic regulatory architecture of muscle-specific gene expression is highly and specifically enriched in muscle stretch/super enhancers. We identified T2D and related trait GWAS SNPs as cis-eQTLs for several genes, including complex transcriptional and splicing regulation of the muscle-specific isoforms of *ANK1* that is associated with SNPs that reside within a muscle stretch enhancer. Together, these studies define links between GWAS SNPs and their target genes in skeletal muscle, providing functional insights with potential precision therapeutic implications for T2D.

## Methods

### Sample recruitment

We attempted to contact still-living FUSION spouses and offspring who participated in FUSION study visits between 1994 and 1998 (ref. 37), individuals who had participated in the population-based Savitaipale Prospective Diabetes Study^38^, the FINRISK 2007 survey, the Dose Responses to Exercise Training (DR's EXTRA) study^39^ and the Metabolic Syndrome in Men (METSIM) study^40^. Additional subjects were recruited by newspaper advertisements. We excluded individuals: (1) with drug treatment for diabetes, (2) with diseases that might be expected to confound the analyses (for example, cancer, skeletal muscle diseases, acute or chronic inflammatory diseases), (3) with diseases that increase haemorrhage risk during biopsy (for example, von Willebrand's disease, haemophilia, severe liver diseases), (4) taking medications that need to be taken daily and increase haemorrhage risk in the biopsies including warfarin (patients on acetosalicylic acid were instructed to stop for 7 days prior to biopsy), (5) taking medications that could confound the analyses (for example, oral corticosteroids, other anti-inflammatory drugs such as 5-ASA, infliximab or methotrexate), and (6) of age <18 years. The study was approved by the coordinating ethics committee of the Hospital District of Helsinki and Uusimaa. A written informed consent was obtained from all the subjects.

### Clinical visit

We performed clinical visits in Helsinki, Savitaipale and Kuopio. 279 individuals participated in both clinical and muscle biopsy visits (see below). The clinical visit included a 2-h, four-point OGTT and other phenotypes measured after a 12-h overnight fast, and health history, medication and lifestyle questionnaires. The clinical visit took place for an average of 14 days before the biopsy visit (90% of clinical visits ≤32 days before biopsy; range 89 days before to 15 days after). We defined glucose tolerance categories of NGT, impaired glucose tolerance (IGT), impaired fasting glucose (IFG) and T2D using World Health Organization (WHO) criteria^41^.

### Phenotype measurements and laboratory analysis

We measured height and weight in light clothing. Waist circumference was measured midway between the lower rib margin and the iliac crest. We determined OGTT plasma glucose (fluoride citrate plasma) concentrations by hexokinase assay (Abbott Architect analyzer, Abbott Laboratories, Abbott Park, IL, USA) and serum insulin by chemiluminescent microparticle immunoassay (Architect analyzer). Glucose and insulin analyses were done at a certified core laboratory at the National Institute for Health and Welfare, Helsinki, Finland.

### Muscle biopsy visit

Biopsies were performed using a standardized protocol and one physician (T.A.L.) trained all doctors performing biopsies. We instructed participants to avoid strenuous exercise for at least 24 h prior to biopsy. Following overnight fast, we obtained ∼250 mg vastus lateralis skeletal muscle using a conchotome, under local anesthesia with 20 mg ml^−1^ lidocaine hydrochloride without epinephrine. Altogether 9 experienced and well-trained physicians collected 331 muscle biopsies in 2009–2013 in 3 different study sites (Helsinki, Kuopio and Savitaipale). Three physicians, one in each site, performed most of the biopsies (237). All physicians were trained to perform the biopsy in an identical manner. The muscle samples were cleaned of blood, fat and other non-muscle tissue by scapel and forceps, rinsed with NaCl 0.9% solution, and frozen in liquid nitrogen. Samples were frozen within 30 s after sampling. Muscle samples were then stored at −80 °C for a duration of 0–4 years before analysis. Overall, the biopsy procedure was well-tolerated. Apart from a few expected cases of bruising, numbness at the biopsy site and vasovagal reactions, there were no clinically significant adverse sequelae.

### RNA isolation and mRNA sequencing

We visually dissected 30–50 mg of each frozen muscle biopsy sample to avoid adipose tissue. Total RNA was extracted and purified with Trizol (Invitrogen, Carlsbad, CA). RNA integrity numbers ranged from 7.2 to 9.4 (median 8.5). To minimize and quantify batch effects, we randomly queued samples for sequencing using a 24-sample barcode-pooling approach and targeted proportional representation of the OGTT states (NGT, IGT, IFG and T2D) in each sequencing batch. External RNA Controls Consortium (ERCC) RNA controls were spiked prior to barcoding to facilitate library quality control (QC). Poly(A)-selected RNA samples were sequenced by the NIH Intramural Sequencing Center (NISC) using the Illumina TruSeq directional mRNA-seq library protocol to a targeted depth of >80 million 100 bp paired-end reads per sample. In total, 279 samples and 7 technical replicates were sequenced in 3,386 read groups on 164 lanes using 6 different HiSeq sequencing machines.

### mRNA-seq processing and QC

We retained RNA-seq reads passing the Illumina chastity filter and mapped reads to a reference sequence composed of ERCC control fragments and all chromosomes and contigs from hg19, excluding alternate haplotypes, replacing chromosome M with the Cambridge Reference Sequence and masking the pseudoautosomal region on chromosome Y. We aligned reads using STAR (version 2.3.1y)^42^ with default parameters and a splice junction catalogue based on Gencode v19 (ref. 43). Duplicate read pairs were retained. Non-uniquely mapping reads and read pairs with unpaired alignments were discarded.

RNA-seq QC was performed at the level of read groups (that is, a library on a lane) using QoRTs^44^. We inspected the comprehensive set of QC metrics generated by QoRTs for outlying libraries, lanes and sequencing runs. We additionally used the 92 ERCC RNA spike-in controls and in-house scripts to assess library quality and batch effects, and to check the accuracy of the strand-specific protocol^45^. This process revealed one outlying library for insert size and a second library with gene body coverage skewed towards the 3′ end, possibly indicating RNA degradation. Both of these libraries were removed from further analyses.

Inspection of read counts revealed systematic batch effects for the proportion of reads mapping to the mitochondrial genome, with up to 52% mitochondrial reads in some sequencing batches. We examined library degradation within affected batches by inspecting gene body coverages for individual highly expressed single-isoform genes and ERCC spike-in transcripts. This did not show any evidence of RNA degradation, but did reveal the presence of systematic batch effects with patterns of read coverage heterogeneity along genes or spike-in transcripts, typically highly similar within batches with noticeable systematic differences between some batches. This result strongly suggests we can correct for these in downstream analyses. Further analysis revealed that batches with high-mitochondrial read fractions had lower estimated RNA fragment lengths.

To address variability in mitochondrial read fraction, we calculated fragments per kilobase transcript per million mapped reads (FPKMs) separately for nuclear genes, mitochondrial genes and ERCC transcripts. PCA on the nuclear genome FPKM matrix and colour labelling by sequencing batch also revealed the presence of systematic batch effects. PCA on the ERCC spike-in FPKM matrix recapitulated these batch effects. In our primary trait-gene expression association analysis, we correct for these observed batch effects. In the cis-eQTL analyses, we correct for these observed batch effects, as well as unknown technical confounders, by using the PEER framework^46^. PCA after PEER correction effectively removed batch effects (Supplementary Fig. 21). We further investigated possible technical confounders in mRNA-seq by checking for sample-specific GC-content biases, which have been reported in early RNA-seq studies. We found no evidence for such effects in our data.

We used verifyBamID^47^ to check for sample swaps and contamination. We asked if reads in the RNA-seq BAM files matched the SNP chip genotype data in transcribed regions for each individual, and determined whether BAM files were contaminated and comprised of reads derived from more than one individual. We identified two pairs of sample swaps which we were able to correct; one of these samples had an estimated contamination of 8% and was excluded. As an additional check for sample swaps and to detect outliers, we verified reported sex by examining expression of the *XIST* gene and mean Y chromosome gene expression. PCA on the FPKM matrix identified four samples as outliers; they were removed from further analyses. One additional sample was an outlier with respect to age (20 years) and was excluded. After all QC exclusions, 271 samples remained and were used for trait-associated expression analyses; of these, 267 had genotype data available and were used for cis-eQTL analyses.

### Expression quantification

To study a wide spectrum of regulatory variation, we performed analyses at three levels: gene, exon and transcript. Definitions for all transcriptome features were based on GENCODE v19 (ref. 43). We counted fragments mapping to genes using htseq-count v0.5.4 (ref. 48) (http://www-huber.embl.de/users/anders/HTSeq/doc/count.html) and calculated FPKM values for each gene. For differential expression analysis and cis-eQTL mapping, we filtered for genes with five or more counts in ⩾25% of samples. We counted reads in exonic parts of genes using dexseq_count v1.0.2 (ref. 49) and calculated exon-level FPKMs for all transcripts in the GENCODE v19 comprehensive annotation. To avoid double-counting of fragments in the quantification of exon abundance, we clipped overlapping read pair mates using the ClipOverlap function of BamUtil (http://genome.sph.umich.edu/wiki/BamUtil:_clipOverlap). We estimated transcript abundance using rSeq (http://www-personal.umich.edu/~jianghui/rseq/) which is based on a Poisson regression model^50^. This model uses information from the insert length distribution inferred using the aligned read fragments which has been shown to help improve estimation. To reduce the number of transcripts per gene to avoid identifiability issues and to restrict analysis to high-confidence transcripts, we estimated transcript expression values for the subset of GENCODE transcripts with the tag ‘basic' in the GTF file.

### Trait–gene expression association

We use ‘trait' to refer to T2D status or a related quantitative trait. First, we describe trait–gene expression analyses with adjustment for known covariates. Second, we describe an analysis that also includes adjustment for unknown factors learned from the gene expression data. Our primary analysis was without adjustment for unknown factors since we were concerned that the unknown factors might include biological signal, as well as expression differences due to technical issues. We compare the results of the two analyses.

### T2D–gene expression association

For individual *i* and gene *j*, let *T*_*i*_ denote T2D status {0=NGT, 1=T2D} and *Z*_*i*_={*Z*_1_,*Z*_2_,….,*Z*_*C*_}^*T*^ the vector of *c* covariates. Let *Y*_*ij*_, denote the rank-based inverse normalized FPKM_*ij*_ where inverse normalization is performed for each gene, randomly breaking ties. We tested for association between *Y*_*ij*_ and T2D status using the linear regression model:


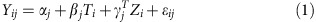


where *α*_*j*_ is the intercept, *β*_*j*_ is the regression coefficient for T2D status on gene *j*, *γ*_*j*_ a vector of coefficients for the covariates and *ɛ*_*ij*_ is a normally distributed error term with mean 0 and variance *σ*^2^. We included as covariates age, sex and experimental batch.

### Quantitative trait–gene expression association

To define a transformed quantitative trait *X*_*i*_, we (1) inverse normalized the raw quantitative trait, (2) adjusted for age, sex and experimental batch by linear regression, and (3) inverse normalized the resulting residuals. To define the gene expression value, *Y*_*ij*_, for each gene we (1) inverse normalized FPKM_*ij*_, (2) performed linear regression of age, sex and experimental batch on the inverse normalized FPKM_*ij*_ and (3) inverse normalized the resulting residuals. We then tested for association between transformed gene expression *Y*_*ij*_ and each transformed quantitative trait *X*_*i*_ using linear regression model:


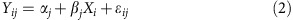


where *β*_*j*_ is the regression coefficient for *X*_*i*_ on gene *j*.

We used FDR^51^ to account for multiple testing and considered as significant associations with FDR≤5%.

### Association analysis adjusting for unobserved confounders

To examine the effect of potential unobserved technical confounders on gene expression–trait association, we modified our definition of transformed gene expression *Y*_*ij*_ by (1) inverse normalizing FPKM_*ij*_ (2) performing factor analysis via PEER^46^,^52^ on the inverse normalized FPKMs specifying from 1 to 15 factors, together with covariates age, sex and experimental batch, and (3) inverse normalizing the residuals. We used the transformed *Y*_*ij*_ to perform differential expression analysis for each trait as above for T2D (equation (1)) and quantitative traits (equation (2)). For each trait, we selected the number of factors that maximized the number of differentially expressed genes. We ran GO term analysis and compared the results to those obtained without adjustment (see below)

### Association analysis adjusting for tissue heterogeneity

The presence of non-muscle cells/tissue within the muscle biopsies may influence the trait and quantitative trait-expression analysis. To investigate this, we estimated tissue heterogeneity, via tissue deconvolution analysis, using the DeconRNASeq R package (v1.8.0)^53^. As a reference transcriptome panel, we used skeletal muscle, adipose, WBC and lymph node transcriptomes from Illumina Body Map 2.0, randomly subsampling reads to an equal amount and calculating FPKMs. For each reference tissue, we calculated the expression specificity index (see below) of each gene and selected the top 500 tissue-specific genes per tissue to use as the reference set. Using the combined tissue-specific gene set, we estimated the tissue heterogeneity of each skeletal muscle biopsy sample. In our samples, we estimated <0.1% adipose contamination, 9–27% WBC, 0–18% lymphocyte and 64–86% skeletal muscle across samples.

To adjust for the effects of non-muscle tissue, we included the estimated percentages of WBCs and lymph as additional covariates in our trait and quantitative trait-expression analysis. We ran GO term analysis and compared the results with those obtained without adjustment (Supplementary Fig. 2).

### GO term enrichment analysis

For each trait, we performed GO term enrichment analysis using RNA-Enrich^6^. For each trait, we define *P* as the signed-log_10_(trait–gene expression *P* value), signed as ‘+' for trait–gene expression association in which higher values of gene expression are associated with T2D, and ‘−' for lower gene expression associated with T2D. We used the logistic regression model


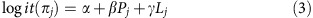


where *π*_*j*_ is the probability of GO term membership for gene *j*, *α* is the intercept, *β* is the regression coefficient for association of GO term membership with *P*, the signed –log_10_(*P* value), and *γ* is the regression coefficient for the GO term membership with *L*, the log_10_(gene length). We include *L* in the model to account for the potential confounding effect of gene length on the enrichment test; longer genes tend to have higher power for expression–trait association, and many GO terms contain set of genes that are substantially longer or shorter than average.

To present the GO term results, for each of the 4 traits tested we retained the 20 most statistically significant GO terms in which genes showed positive association with the trait and the 20 most significant GO terms in which genes showed negative association with the trait. Within each trait and direction of association, we ranked the GO terms from 1 (most significant) to 20. We combined the 40 × 4=160 GO terms and assigned the lowest rank when GO terms appeared more than once on the list. We pruned redundant GO terms from the combined lists of GO terms, preferentially retaining GO terms with lower ranks^54^. We hierarchically clustered the pruned GO terms based on the regression coefficients (*β*'s) from equation (2) from the four traits using complete linkage and the Euclidean distance measure in four-dimensional space.

To assess the robustness of our findings, we repeated RNA-Enrich^6^ analysis using the PEER factor adjusted trait–gene expression association results (see above). Results were very similar.

### Sample and genotype QC

We extracted DNA from blood. DNA samples were genotyped at the Genetic Resources Core Facility (GRCF) of the Johns Hopkins Institute of Genetic Medicine on the HumanOmni2.5-4v1_H BeadChip array (Illumina, San Diego, CA, USA). We mapped the Illumina array probe sequences to the hg19 genome assembly using the Burrows-Wheeler Aligner (BWA)^55^. We excluded SNPs with probe alignment problems, known variants in the 3′ end of probes, call rates <95%, minor allele count (MAC) <1 or Hardy–Weinberg equilibrium *P* value <10^−6^, leaving 1,642,012 SNPs for subsequent analysis. All alleles were oriented relative to the reference.

Of the 271 individuals that passed RNA-seq QC, we genotyped 267 samples, of which all were successful, with minimum call rate >98.7%. Based on 10 duplicate samples (from a larger set of genotyped samples), overall genotype concordance was 99.993%. We identified two unexpected pairs of first-degree relatives using KING (http://people.virginia.edu/~wc9c/KING/). Each was an NGT-IGT pair; from each pair we excluded the NGT participant. We performed principal components analysis using SMARTPCA^56^ on 156,416 SNPs with MAF>5% and in near linkage equilibrium (*r*^2^<0.2), after excluding SNPs from regions of high LD^57^. No population outliers were identified.

### Imputation

We performed SNP imputation using a two-step strategy^58^. As reference panel we used the haplotypes from 2,737 European individuals sequenced in the GoT2D project. To improve phasing quality given the small target sample set, we pre-phased our 267 individuals together with the GoT2D reference panel samples using ShapeIT version 2 (https://mathgen.stats.ox.ac.uk/genetics_software/shapeit/shapeit.html). We then imputed genotypes with Minimac2 (ref. 59). For chromosome X, we performed pre-phasing and imputation separately for the pseudo-autosomal and non-pseudo-autosomal regions. For cis-eQTL analysis, we included 8,406,237 variants with imputation quality *r*^2^>0.3 and MAC>5.

### cis-eQTL analysis

We performed cis expression quantitative trait (eQTL) analysis of SNPs within 1 Mb of the most upstream TSS of each gene using Matrix eQTL^60^, separately at the levels of gene, exon and transcript isoforms. To generate the gene expression value *Y*_*ij*_, we (1) inverse normalized FPKM_*ji*_ (2) performed factor analysis via PEER on the inverse normalized FPKM (separately for genes, exons and transcript isoforms; specifying from 1 to 100 factors; and including age, sex, OGTT status, the top 2 genotype-based principal components and experimental batch as covariates in the model, and (3) inverse normalized the resulting residuals. We used the linear regression model with an additive genetic effect


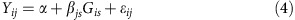


where *G*_*is*_ is the imputed allele count for SNP *s* for individuals *i*, and *β*_*js*_ is the regression coefficient of the imputed allele count for SNP *s* on transformed gene expression *Y*_*ij*_.

We used FDR^51^ to account for multiple testing and considered as significant associations with FDR≤5%.

We present results based on 60 PEER factors since we expect that removing technical and biological variation will increase power to detect cis-eQTLs and we observed very little increase in the number of cis-eQTLs (FDR≤5%) for >60 factors^17^ (Supplementary Fig. 22).

### Comparison to GTEx skeletal musclegenic cis-eQTL results

We downloaded the entire set of skeletal muscle SNP–gene association tests (V6) for 361 GTEx skeletal muscle samples^5^ from the GTEx portal (www.gtexportal.org). We called GTEx SNP–gene association tests with FDR≤5% as significant. About 95,528,846 SNP–gene pairs (19,038 genes and 6,694,033 SNPs) were tested in common in the 2 studies and had concordant alleles. We oriented the association results in each study to the same effect allele.

### Genic cis-eQTL for GWAS variants for T2D and related traits

We compiled a list of 225 GWAS variants for T2D, fasting glucose, fasting insulin and 2-h glucose, each with and without adjustment for BMI, and for fasting proinsulin, from the NHGRI GWAS catalogue^61^ and carried out manual curation of the literature to create a comprehensive list that was up-to-date as of May 2014. We crossed this list of 225 GWAS variants with our list of gene-based cis-eQTL results, resulting in a total of 220 SNPs and 4,545 tested GWAS SNP–gene pairs. To identify GWAS variant cis-eQTLs that are likely independent of other stronger cis-eQTLs for the same gene, and to calculate a conditional-analysis-based FDR, we performed iterative conditional analysis on each of the 4,545 SNP–gene pairs. For each such pair, we predicted *Y*_*ij*_ (as defined for cis-eQTL analysis) starting with the GWAS SNP genotype in the model, and then performed step-wise forward selection of SNPs within 1 Mb of the most upstream TSS, with a stopping threshold of a *P* value of 0.0019 (corresponding to the *P* value threshold for gene-based cis-eQTLs with FDR<5%).

### ASE measurements

Our goal was to quantify ASE at each protein-coding SNP. Based on the calls from our DNA genotyping and imputation, for each individual we identified all sites at which a SNP in a gencode V19 annotated protein-coding exonic region was called as a heterozygote. At these sites, we quantified the strand-specific read coverage using samtools mpileup (version 0.1.18) to process the aligned RNA-seq read BAM files. We required a minimum mapping quality of 255, minimum base quality of 20 and reads mapped in a proper pair. We excluded reads that failed vendor quality checks or that were not the primary alignment. ASE was defined as the reference allele read count divided by reference+alternate read count, termed fracRef.

### ASE filtering to adjust for read mapping bias

We implemented multiple filtering steps to identify and exclude SNPs susceptible to mapping errors that could bias ASE quantification. To identify SNPs with mapping biases, we simulated reads as previously described^9^, except we used 101 bp reads, and after mapping the simulated reads excluded SNPs with a total simulated coverage of <193 and >202. We excluded SNPs based on additional filters. Although mono-allelic expression occurs at some sites, we required representation of both alleles in each SNP per individual by requiring 0.01<fracRef<0.99. In addition, we excluded SNPs in regions blacklisted by the ENCODE Project Consortium^62^ because of poor mappability or the presence of collapsed repeat regions^10^. We excluded any SNP within 101 bp of an indel greater than 4 bp or overlapping an indel of any length.

### ASE statistical analysis

fracRef varies systematically by the reference and alternate allele of the SNP. As previously described^9^, starting with the filtered SNP list, for each sample, for each SNP reference and alternate allele pair (for example, AG and GA are separate allele pairs), we estimated the expected fracRef. The sample-specific and allele-pair-specific expected fracRef was calculated as the sum of the reference allele counts divided by the sum of the total allele counts across all SNP of a given reference and alternative pair for an individual. To prevent SNPs with high coverage from biasing the estimated fracRef, SNPs with read count coverage in the top 25th percentile were down-sampled to 30 × coverage and the down-sampled reference allele and total count were used. For each individual and for each SNP, we performed a two-sided binomial test, using the observed sample-specific or allele pair-specific fracRef as the true fracRef under the null hypothesis of no ASE. Of the set of SNPs for each individual, we call ASE significant if the Storey's FDR *q*-value≤0.05. The forward and reverse stranded ASE quantifications were combined after filters and before statistical analysis for accurate sample-specific adjusted expectation calculation. To increase power to detect ASE, at each SNP that meets the above thresholds and occured in ⩾10 samples, we combined the binomial test *P* values using Fisher's combined probability test.

### Analysis of muscle-specific expression

We used an information theory approach^13^,^14^ to score genes based on muscle expression level and specificity relative to the panel of 16 diverse Illumina Human Body Map 2.0 tissues. We first calculated expression (*x*) in FPKM values for all Gencode v19 genes across each of the 16 tissues in the Body Map 2.0 data. We calculated the relative expression of each gene (*g*) in skeletal muscle compared with all 16 tissues (*t*) as *p:*


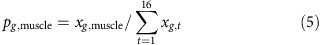


We next calculated the entropy for expression of each gene across all 16 tissues as *H:*


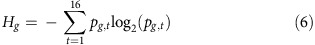


Following previous studies^13^,^14^, we defined muscle tissue expression specificity (*Q*) for each gene as:


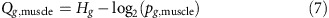


To aid in interpretability, we divided *Q* for each gene by the maximum observed *Q* and subtracted this value from 1 and refer to this new score as the mESI:


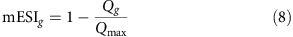


mESI scores near zero represent low and/or ubiquitously expressed genes, and scores near 1 represent genes that are highly and specifically expressed in skeletal muscle. We note that although these calculations were performed using expression measured by FPKM, we obtained similar results when using transcripts per million.

### Chromatin state analyses

We performed read mapping and integrative chromatin state analyses as in our previous report^15^. Chromatin states were learned jointly by applying the ChromHMM (version 1.10) hidden Markov model (HMM) algorithm at 200 bp resolution to six data tracks (Input, K27ac, K27me3, K36me3, K4me1, K4me3) from each of the cell or tissue types^11^. We collected cell or tissue ChIP-seq reads from a diverse set of publically available data^11^,^15^,^63^,^64^ representing 31 cells/tissues. We ran ChromHMM with a range of possible states, and settled on an 11-state model as it accurately captured information from higher state models and provided sufficient resolution to identify biologically meaningful patterns in a reproducible way. This process was similar to our previous analysis^15^. To determine how our learned states relate to previously published states from nine cell types^11^, we performed enrichment analyses comparing our states with the published states in each cell type (Supplementary Fig. 6). We also performed gene body feature overlaps, and TSS proximity analyses^11^. This process led to a clear state assignment (Supplementary Fig. 6), which we used for all subsequent analyses.

We measured enrichment of cis-eQTLs at various levels of significance (FDR thresholds of 5, 1 and 0.1%) to overlap chromatin states by retaining the single best (lowest *P* value) cis-eQTL per gene, and using the GREGOR tool^12^ to calculate enrichment relative to MAF, TSS-distance and number of LD neighbours-matched null SNP sets. The enrichment trends were consistent across the different FDR thresholds, with more stringent sets having slightly more pronounced trends. We report here the results for the FDR=0.1% set. We used the following GREGOR parameters, which are what was reported in the original publication: *r*^2^ threshold=0.8, LD window size=1 Mb and minimum neighbour number=500.

### ATAC-seq sample processing

ATAC-seq libraries were prepared using a modified protocol based on previous studies^20^. Frozen human skeletal muscle tissue (Zen-bio, Durham, NC USA) was cut into a 100-mg piece and disrupted using liquid nitrogen and a CellCrusher (Cellcrusher, Cork Ireland). Nuclei were isolated by placing disrupted tissue into ice cold nuclei isolation buffer (NIB) (20 mM Tris-HCl, 50 mM EDTA, 5 mM Spermidine, 0.15 mM Spermine, 0.1% mercaptoethanol, 40% Glycerol, pH 7.5). The solution was then filtered through a Miracloth (Calbiochem, San Diego, CA USA) and centrifuged at 1,100*g* for 10 min at 4 °C. The resultant nuclei pellet was washed with NIB containing 0.5% Triton-X and RSB buffer (10 mM Tris-HCl, 10 mM NaCl, 3 mM MgCl2, pH 7.4). The nuclei pellet was resuspended in 50 μl RSB buffer, and 1:10 (10 mg total tissue) and 1:50 (2 mg total tissue) dilutions were made. The transposition reactions were performed using a homemade Tn5 preparation (courtesy of the Jacob Kitzman laboratory)^20^. The DNA was then purified using the Qiagen MinElute PCR Purification kit (cat. No. 28004). Next, the libraries were PCR amplified to 9 cycles for the 1:10 dilution and 11 cycles for the 1:50 dilution. A final clean-up was performed with the Qiagen MinElute PCR Purification kit and samples were sent to bioanalyzer for QC.

### ATAC-seq analyses

We sequenced each ATAC-seq library to a depth of >48M reads using a 50 bp paired-end read layout on an Illumina HiSeq. Adapter sequence was trimmed from the raw ATAC-seq reads using a tool we developed based on the one used in the original ATAC-seq paper^20^. Trimmed reads were then aligned to the hg19 reference with BWA aln (http://bio-bwa.sourceforge.net/) version 0.7.12. Duplicate alignments were marked with Picard Tools (https://broadinstitute.github.io/picard/) version 1.131 and discarded for further analyses. We then filtered for properly paired alignments and for reads uniquely mapped with high quality to autosomal references, using samtools (https://samtools.github.io/) version 1.2 and flags ‘-f 3 -F 4 -F 8 -F 256 -F 1024 -F 2048 -q 30'. Finally, we called peaks using MACS2 (https://github.com/taoliu/MACS/) version 2.1.0, with flags ‘-g hs --nomodel --shift -100 --extsize 200 -B --broad --keep-dup all' and retained peaks that met a 5% FDR. For comparative purposes, we performed the same read trimming, alignment, filtering and peak calling steps on publicly available ATAC-seq data in GM12878 (ref. 20) and adipose tissue^21^.

To detect potential TFBSs using both reference and alternate alleles, we extracted the alternate allele from biallelic SNPs and short indels from 1000 Genomes phase 3 (release v5) along with 29 bp of flanking sequence from the hg19 human reference on each side. We scanned the reference and these extracted alternate allele sequences with position weight matrixes (PWMs) from ENCODE^65^, JASPAR^66^ and Jolma *et al*.^67^ using FIMO^68^. FIMO was run using nucleotide frequencies from the hg19 reference (40.9% GC), and the default *P* value cutoff (10^−4^).

ATAC-seq footprints were called using CENTIPEDE^22^. Briefly, for each PWM scan result, we generated a strand-specific (relative to the motif orientation) 5-bp resolution matrix encoding the number of Tn5 integration events in a region±100 bp from each motif occurrence. We split the ATAC-seq signal into three different categories based on the diverse fragment length distribution: 36–149 bp, 150–324 bp and 325–400 bp. A motif occurrence was considered bound if the CENTIPEDE posterior probability was >0.99 and its coordinates were fully contained within an ATAC-seq peak. We only considered footprints for motifs that were significantly enriched in our data, using an enrichment approach as previously described^69^.

### Electrophoretic mobility shift assay

EMSAs were performed as previously described^70^. Briefly, biotin-end-labelled complementary oligonucleotides were designed (Integrated DNA Technologies, Coralville, IA USA) around the variant rs508419 (5′- AGGATAGG[T/C]GAGAGGCC -3′). Nuclear protein extract from human SkMC, myoblasts (HSMM) (Lonza, Walkersville, MD USA) and mouse muscle myoblasts (C2C12) (ATCC, Manassas, VA USA) was prepared using the NE-PER Extraction Kit (Thermo Scientific, Waltham, MA). The LightShift Chemiluminescent EMSA Kit (Thermo Scientific) was used following the manufacturer's protocol with binding reactions comprising 1 × binding buffer, 1 microgram poly(dI-dC), 6–10 μg nuclear extract and 400 fmol of labelled probe. Reactions were incubated at room temperature for 30 min. Competition reactions had 25–40-fold excess of unlabelled oligonucleotides for either allele. For supershift assays, 1.5–2 μg TR4 antibody (JD Engel Lab, University of Michigan) was added to the reaction and incubated 30 min at room temperature before addition of the labelled probe. EMSAs were repeated and yielded comparable results.

### Data availability

RNA-seq, ATAC-seq and genotype data for all samples used in this paper have been deposited in dbGaP with the accession code phs001068.v1.p1 and are available via the repository's data access request procedures.

## Additional information

**How to cite this article:** Scott, L. J. *et al*. The genetic regulatory signature of type 2 diabetes in human skeletal muscle. *Nat. Commun.* 7:11764 doi: 10.1038/ncomms11764 (2016).

## Supplementary Material

Supplementary Information Supplementary Figures 1-22, Supplementary Tables 1-2

Supplementary Dataset 1 Detection of independent genic eQTLs associated with T2D and related traits. T2D and related trait GWAS and candidate gene-associated variants were tested for association with genes whose most distal TSS was within 1Mb of the variant. ^a^We use GWAS to denote GWAS or candidate gene studies; ^b^Risk/trait increasing allele (set to risk/trait increasing allele for majority of traits when trait coefficient directions are not concordant); ^c^T2D Odds Ratio (OR) or trait effect size for GWAS risk or trait increasing allele; ^d^eQTL effect for GWAS risk or trait increasing allele; ^e^decile 10 denotes most muscle specific expression, NA denotes insufficient expression in the Illumina Body Map tissues to estimate specificity; ^f^Table contains all results with GWAS SNP eQTL q-value

## Figures and Tables

**Table 1:** Detection of independent genic cis-eQTLs associated with T2D and related traits.

GWAS locus gene name*	GWAS trait (s)*	GWAS SNP*	GWAS risk/higher trait level allele*	GWAS OR/ effect size*†	**eQTL effect size**‡	**eQTL gene**	**MESI decile**§	**eQTL** ***q*****-value**	**eQTL conditional** ***q*****-value**||
*ERAP2*	2-h glucose	rs1019503	A	0.063	1.16	*ERAP2*	3	2.1 × 10^−62^	7.0 × 10^−84^
*ERAP2*	2-h glucose	rs1019503	A	0.063	−0.97	*LNPEP*	7	8.1 × 10^−36^	7.8 × 10^−39^
*ERAP2*	2-h glucose	rs1019503	A	0.063	−0.90	*CTD-2260A17.2*	7	1.1 × 10^−28^	4.4 × 10^−33^
*AMT*	Fasting glucose	rs11715915	C	0.012	−0.61	*AMT*	1	1.3 × 10^−10^	5.8 × 10^−32^
*ANK1*	T2D	rs515071	C	1.18	1.01	*ANK1*	10	1.9 × 10^−19^	2.0 × 10^−24^
*ANK1*¶	T2D	rs516946	C	1.09	1.01	*ANK1*	10	1.9 × 10^−19^	2.0 × 10^−24^
*FADS1, FADS1-2-3*	Fasting glucose	rs174550	T	0.022	0.77	*FADS1*	3	4.2 × 10^−18^	3.2 × 10^−20^
*POU5F1/TCF19*	T2D	rs3132524	G	1.07	−0.84	*CCHCR1*	9	4.2 × 10^−17^	1.2 × 10^−19^
*POU5F1/TCF19*	T2D	rs3132524	G	1.07	0.79	*HCG27*	2	4.6 × 10^−15^	1.9 × 10^−14^
*AMT*	Fasting glucose	rs11715915	C	0.012	−0.68	*NICN1*	5	1.6 × 10^−13^	1.4 × 10^−13^
*JAZF1*	T2D	rs849135	G	1.11	−0.38	*JAZF1*	4	5.3 × 10^−4^	5.6 × 10^−11^
*KCNJ11*	T2D	rs5215	C	1.07	−0.33	*ABCC8*	NA	1.5 × 10^−2^	1.4 × 10^−10^
*GPSM1*	T2D	rs11787792	A	1.15	0.38	*GPSM1*	1	1.9 × 10^−3^	6.7 × 10^−10^
*PROX1*	Fasting glucose	rs340874	C	0.021	0.49	*PROX1-AS1*	3	1.3 × 10^−6^	7.5 × 10^−10^
*PROX1*	T2D	rs340874	C	1.07	0.49	*PROX1-AS1*	3	1.3 × 10^−6^	7.5 × 10^−10^
*ZFAND3*	T2D	rs9470794	C	1.12	0.90	*ZFAND3*	9	5.1 × 10^−8^	1.4 × 10^−9^

2-h, 2 hour; eQTL, expression quantitative trait locus; GWAS, genome-wide association studies; SNP, single-nucleotide polymorphism; T2D, type 2 diabetes; TSS, transcription start site.

T2D and related trait GWAS and candidate gene-associated variants were tested for association with genes whose most distal TSS was within 1 Mb of the variant.

^*^We use GWAS to denote GWAS or candidate gene studies.

^†^T2D odds ratio (OR) or trait effect size for GWAS risk or higher trait level allele.

^‡^eQTL effect for GWAS risk or higher trait level allele.

^§^Decile 10 denotes most muscle-specific expression, NA denotes insufficient expression in the Illumina Body Map tissues to estimate specificity.

^||^15 most significant conditional cis-eQTL results (for the 4,545 tested GWAS-SNP–gene pairs) that also have an cis-eQTL *q*-value<0.05 (genome-wide).

^¶^See Supplementary Data 1 for *ANK1* SNP rs515071 (high *r*^2^ with rs516946).
