# Supplementary material for: The genetic regulatory signature of type 2 diabetes in human skeletal muscle
Source: Nat Commun. 2016 Jun 29;7:11764. doi: 10.1038/ncomms11764 (PMC4931250; doi:10.1038/ncomms11764)
Supplement: Supplementary Information — Supplementary Figures 1-22, Supplementary Tables 1-2 [file ncomms11764-s1.pdf]

Supplementary Figures

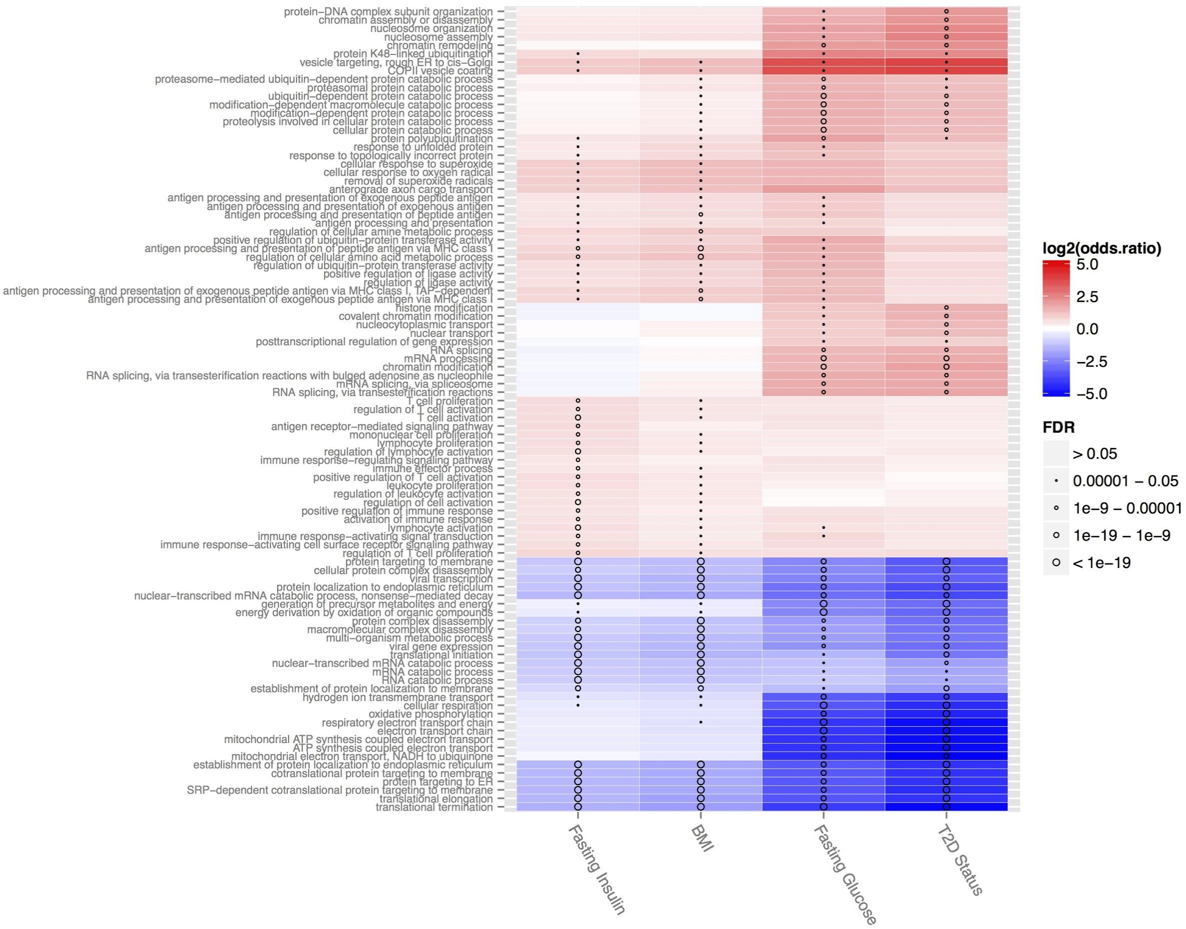

Supplementary Figure 1. Heatmap of GO terms for differentially expressed genes. The terms were hierarchically clustered using the GO term enrichment beta. Darker red, higher positive gene expression-trait association; darker blue, stronger negative association. Larger circle sizes represent more significant GO terms.

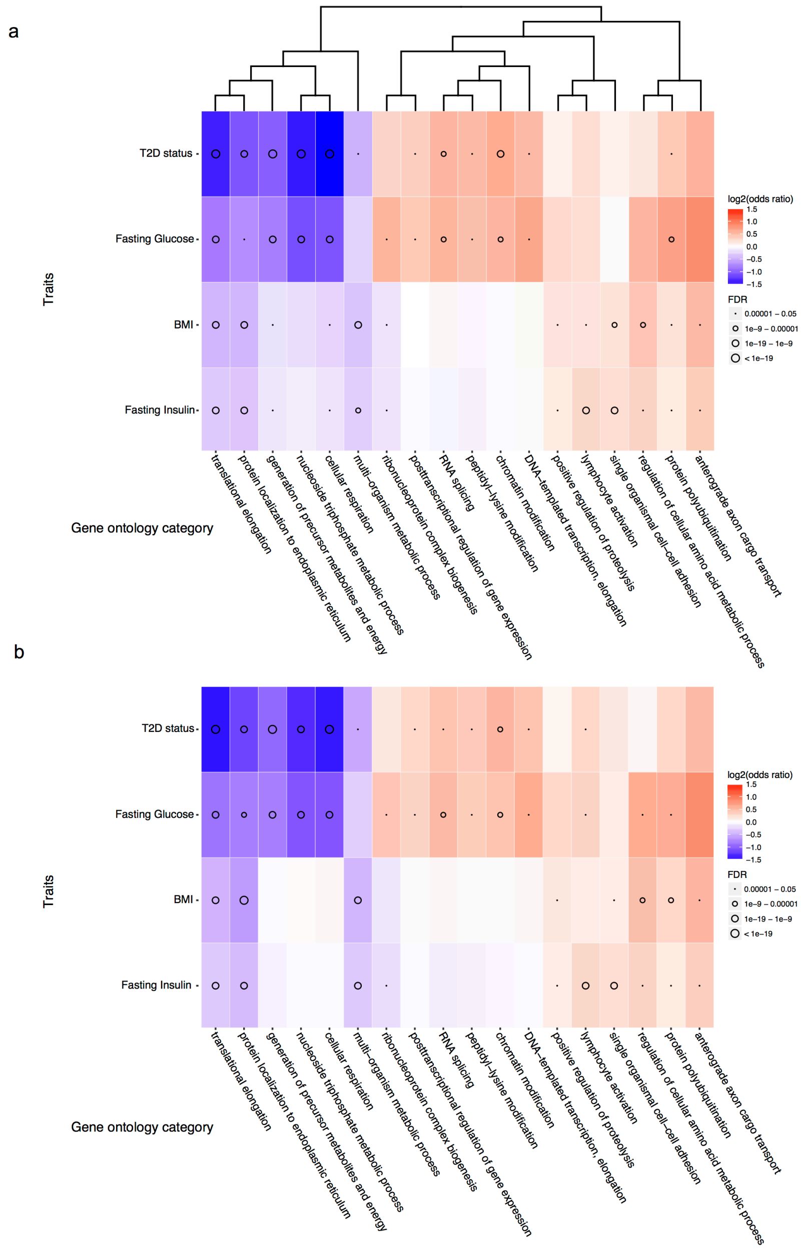

Supplementary Figure 2. Heat maps for GO terms for differential expression analyses without (a) (as in Figure 1c) and with (b) adjusting for fraction white blood cell and fraction lymph from the muscle tissue heterogeneity deconvolution analysis (see Methods). To facilitate comparison between the results of the two analyses, we show the same go terms in (b) as in (a). For each GO term and trait we see similar patterns of higher or lower gene expression with the trait for analyses with and without adjustment for muscle cell heterogeneity. The strength of associations shows some variability between the two analyses.

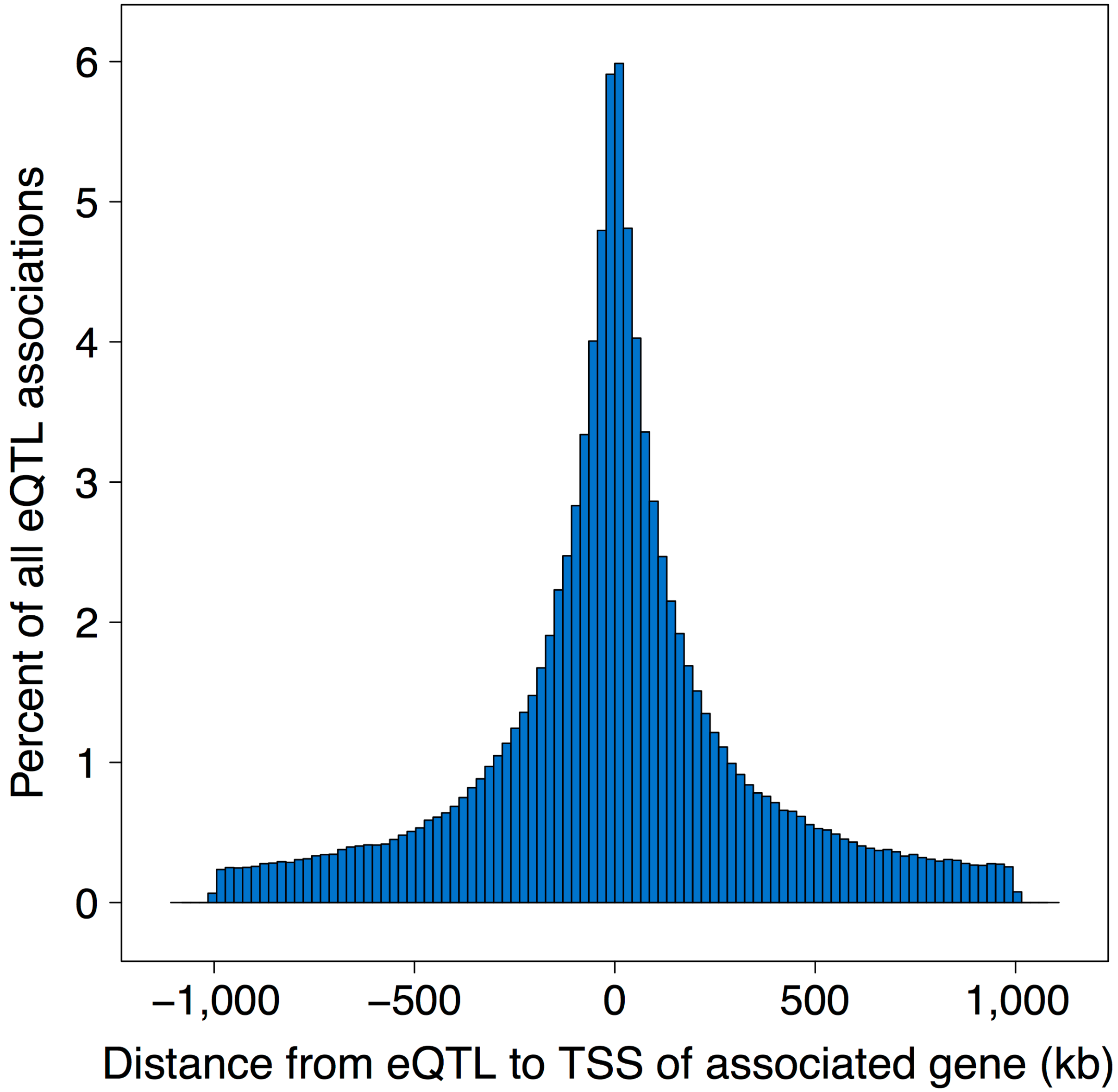

Supplementary Figure 3. Distance (kb) from the cis-eQTL to transcription start site (TSS) of the associated gene.

a

	Significant ASE	Not significant ASE	Not tested for ASE	Total
Significant eQTL	6,830	1,650	5999	14,479
Not significant eQTL	231	112	627	970
Not tested for eQTL	345	62		407
Total	7,406	1,824	6,626	15,856

b

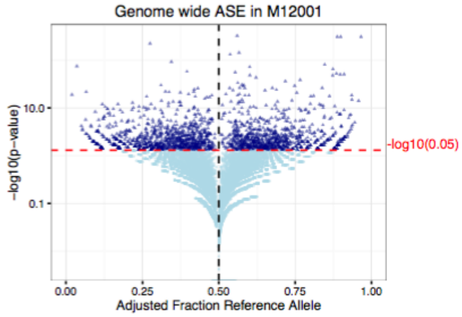

Supplementary Figure 4. Allelic bias in RNA-seq data and comparison with eQTL results. (a) Comparison of eQTL and ASE results in protein coding genes on the autosomes. To be tested for ASE the gene must have at least one SNP that passes filters, has ≥ 30x coverage, and is present in ≥ 10 samples. Significance is determined by Fisher's Combined Probability test and Storey's FDR correction (q-value ≤ 0.05). 94.57% of genes tested for an eQTL have a significant eQTL and 80.24% of genes tested for ASE have significant ASE suggesting almost all genes have genetic regulation. 407 genes were tested by ASE but not for an eQTL and 84.8% had significant ASE. (b) Volcano plot of genome-wise ASE in one muscle sample, M12001. The adjusted fraction reference allele uses a sample specific, allele pair specific expectation to adjust for residual reference allele alignment bias after filtering steps. Reference + Alternate allele count is restricted to those sites with ≥ 30x coverage.

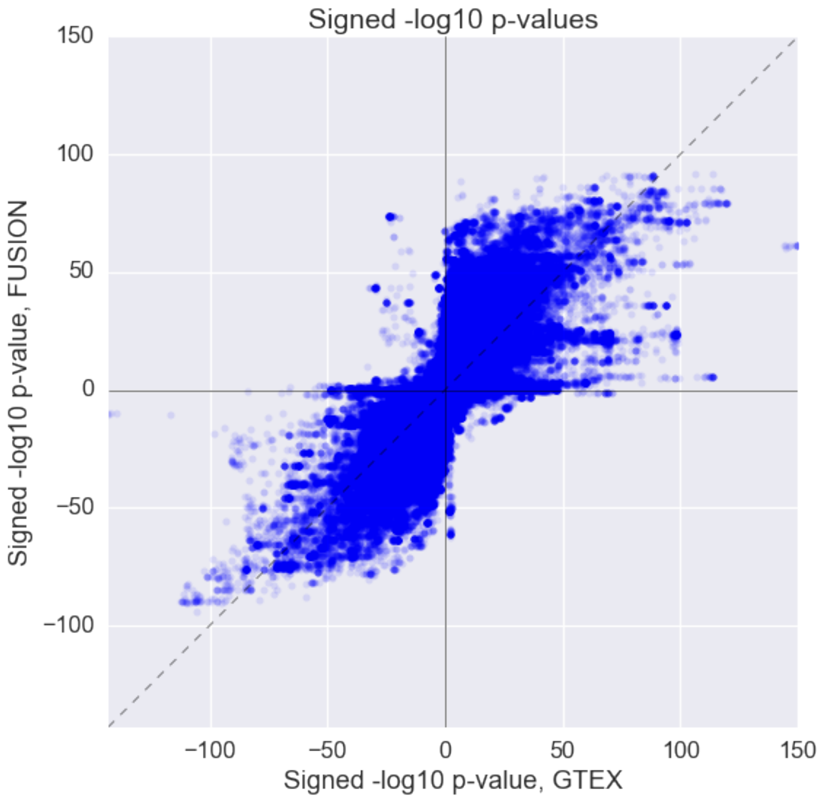

Supplementary Figure 5. Consistency in FUSION (N=267 skeletal muscle samples) and GTEx (N=361 skeletal muscle samples) cis-eQTL results. Here we show the signed (based on the estimated regression coeffcient keyed to the same allele) $\log_{10}$(P-values) for the 95.5M matching cis-eQTL results across the studies. Note that the large majority of results have a consistent direction of effect.

a

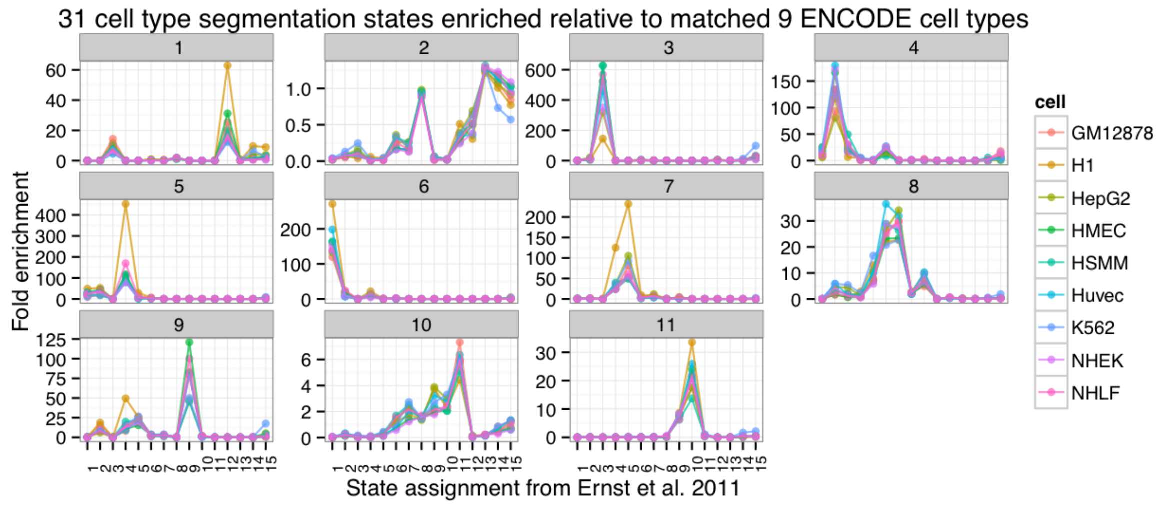

b

original_state	new_state	state_name
1	12	polycomb_repressed
2	13	heterochromatin_low
3	3	inactive_poised_promoter
4	2	weak_promoter
5	4	strong_enhancer
6	1	active_promoter
7	5	strong_enhancer
8	7	weak_enhancer
9	9	transcription_transition
10	11	weak_transcribed
11	10	transcription_elongation

c

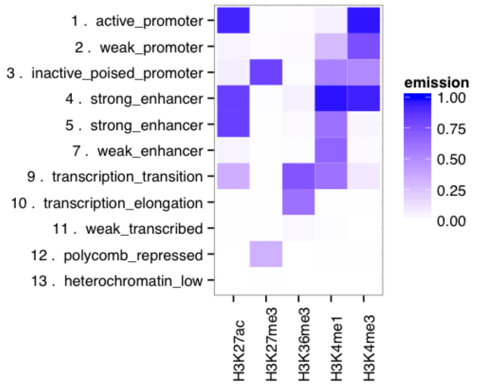

Supplementary Figure 6. Original state assignments from our ChromHMM run (in each facet) were compared to chromatin state assignments in the same cell types published by Ernst *et al*. 2011 (a). Based on enrichment with previously published chromatin state calls, we re-assigned our states (b). The emission probabilities of our re-assigned chromatin state model are represented in (c).

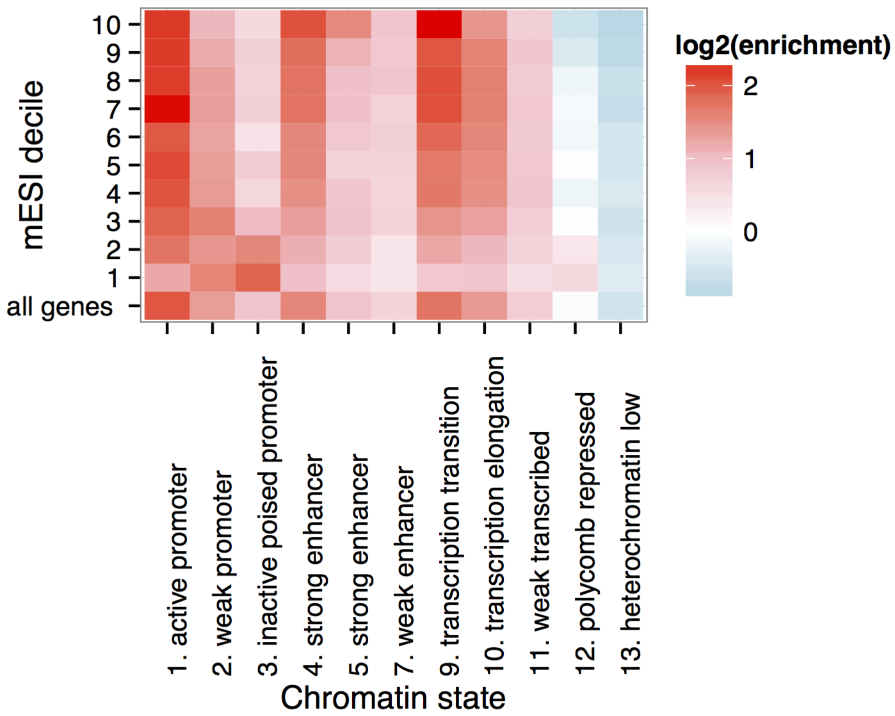

Supplementary Figure 7. Enrichment of 0.1% FDR cis-eQTL in different skeletal muscle chromatin states (x-axis) using either all genes or genes split by mESI decile (y-axis). Note that the level of enrichment increases with mESI decile for the enhancer states and decreases for the polycomb repressed states.

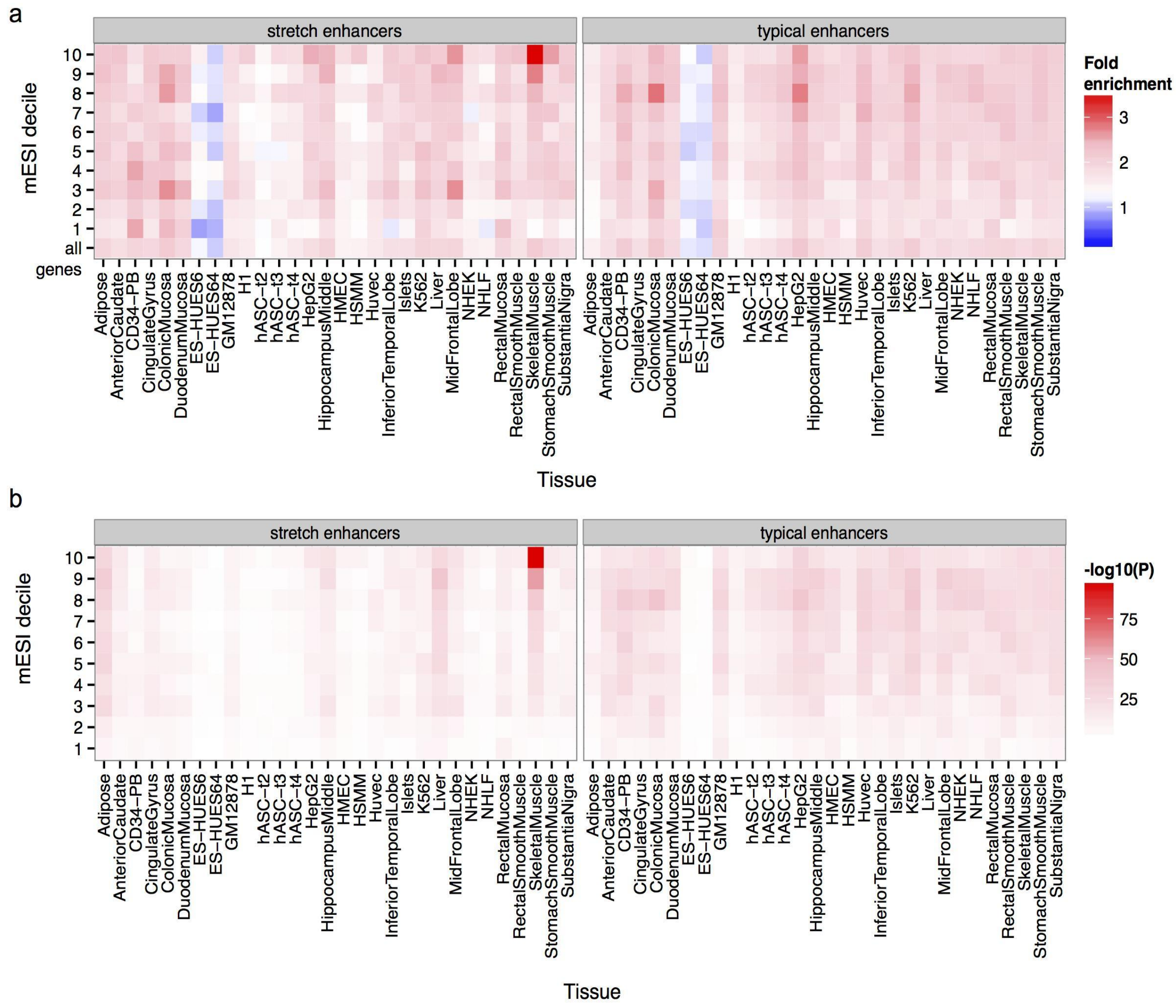

Supplementary Figure 8. Stretch and typical enhancer fold enrichment (a) and significance (b) for overlapping eQTL from difference mESI bins or all genes across multiple cell/tissue types. For skeletal muscle there are 15,007 stretch and 54,878 typical enhancers.

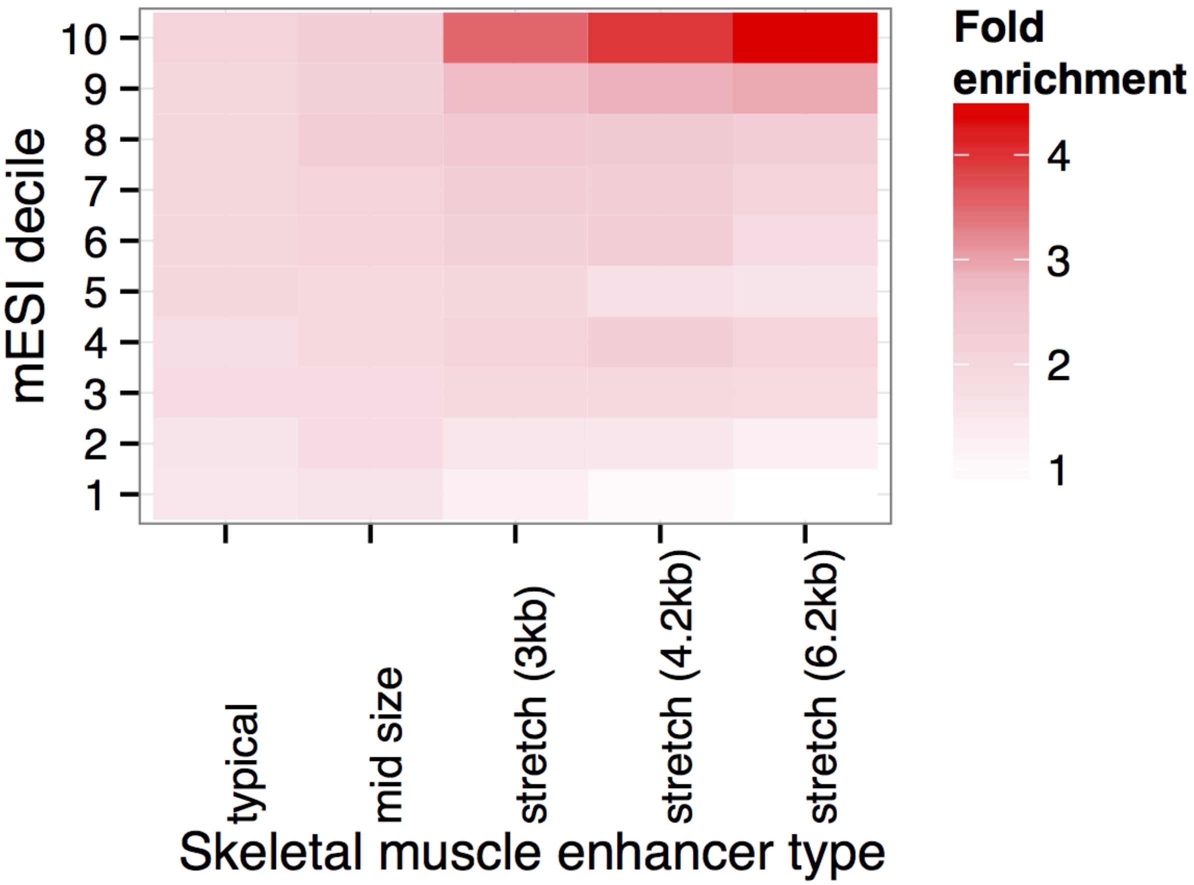

Supplementary Figure 9. Skeletal muscle stretch, typical, and mid-size enhancer fold enrichment for overlapping skeletal muscle eQTL from different mESI bins. Stretch enhancer thresholds of 3.0kb, 4.2kb, and 6.2kb correspond to the previously defined thresholds of the top 10%, 5%, and 1% of enhancer lengths, respectively. Note that the enrichment trend is consistent regardless of the stretch enhancer threshold used. Typical (≤800bp; median length) and mid-size (>800bp and <3kb) enhancers are shown as controls and do not have progressively increasing enrichment trends like stretch enhancers.

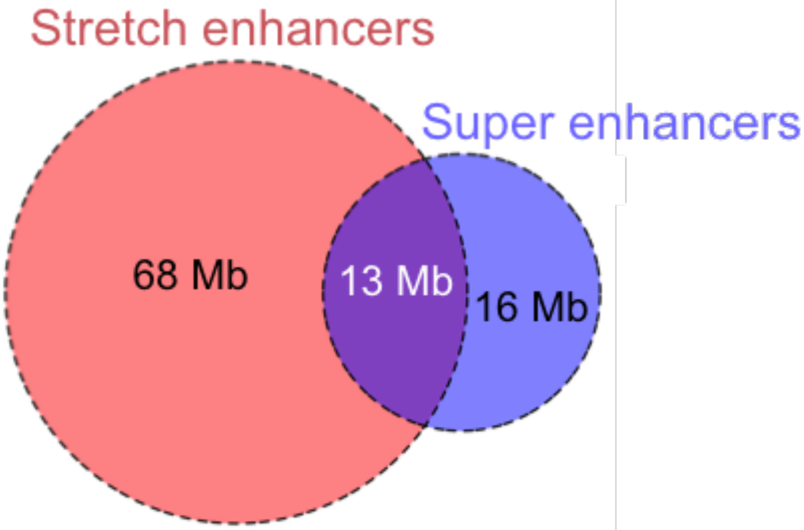

Supplementary Figure 10. Skeletal muscle stretch (default 3.0kb threshold) and super enhancer base pair level overlap comparison.

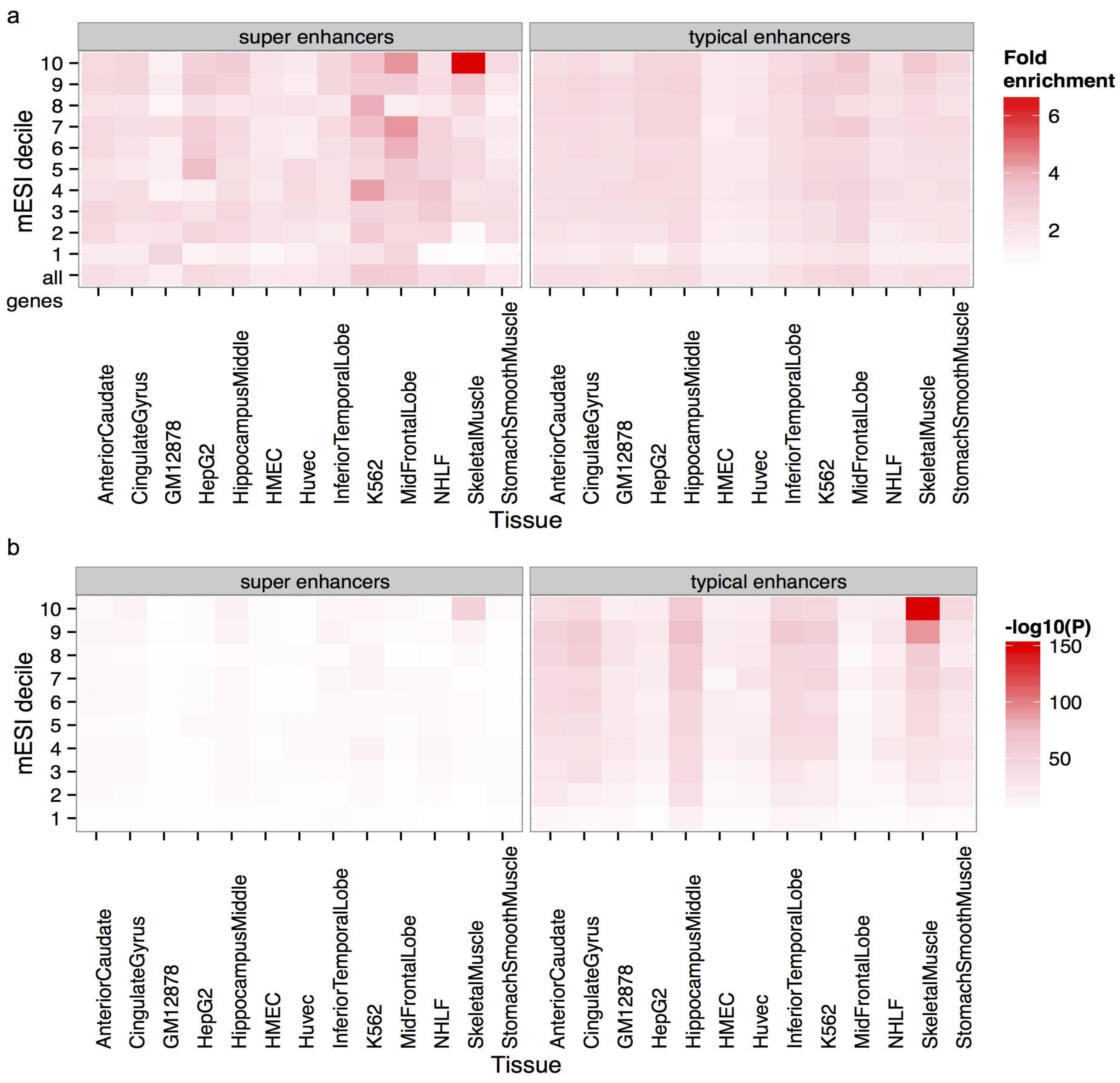

Supplementary Figure 11. Super and typical enhancer (based on super enhancer definition) fold enrichment (a) and significance (b) for overlapping eQTL from difference mESI bins or all genes across multiple cell/tissue types. For skeletal muscle there are 618 super and 18,620 typical enhancers. This large difference in N allows for the skeletal muscle typical enhancers to have more significant p-values (b) even though the enrichment is lower (a) compared to skeletal muscle super enhancers.

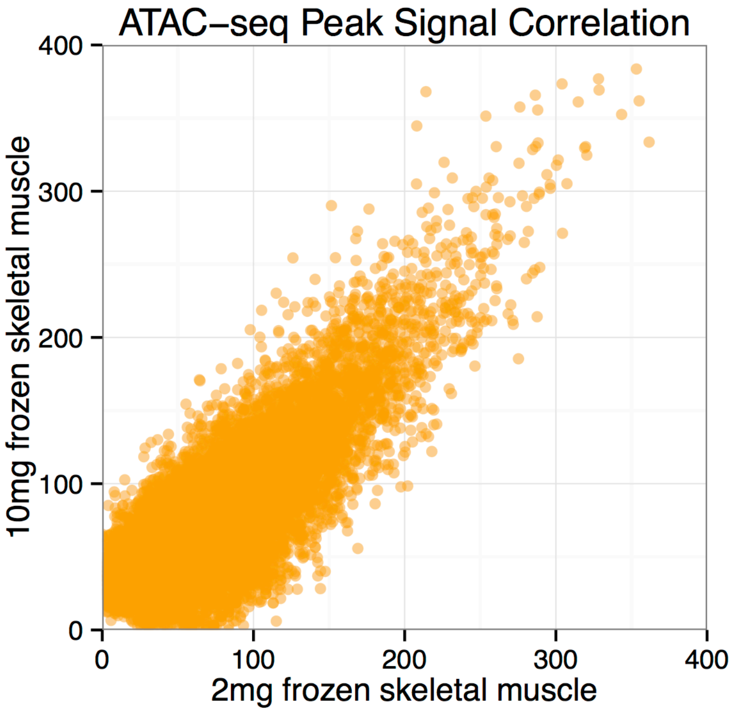

Supplementary Figure 12. Signal correlation across frozen skeletal muscle ATAC-seq 10mg and 2mg input replicates. Peaks were called on individual replicates using MACS2 (see Methods) and then merged across each. Signal within each merged peak is quantified in RPKM units. Pearson's R = 0.815.

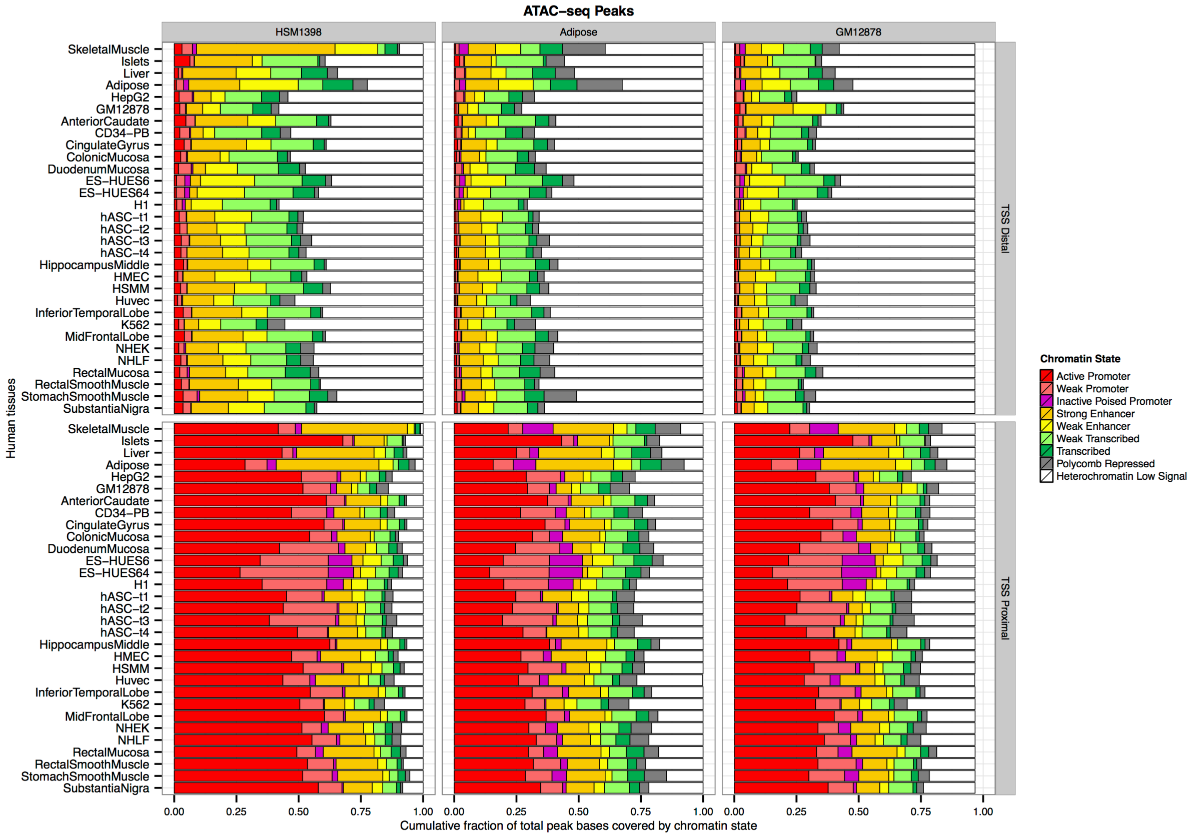

Supplementary Figure 13. Comparison of ATAC-seq peak calls in three cell types (columns) with chromatin states across diverse tissues (y-axis). Skeletal muscle (sample = HSM1398) combined replicate ATAC-seq peak calls (left column) show enrichment for skeletal muscle active chromatin states, which is more pronounced at TSS-distal (>5kb from TSS) regions. Adipose (middle column) and GM12878 (right column) ATAC-seq peak calls show similar trends with chromatin states from matched tissues.

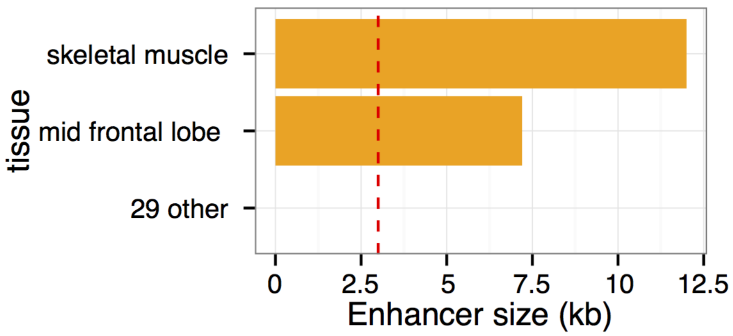

Supplementary Figure 14. Enhancer length overlaps at the T2D GWAS tag SNP rs516946, which is the best cis-eQTL SNP for *ANK1*. The vertical dashed red line represents the default 3kb length threshold for calling stretch enhancers.

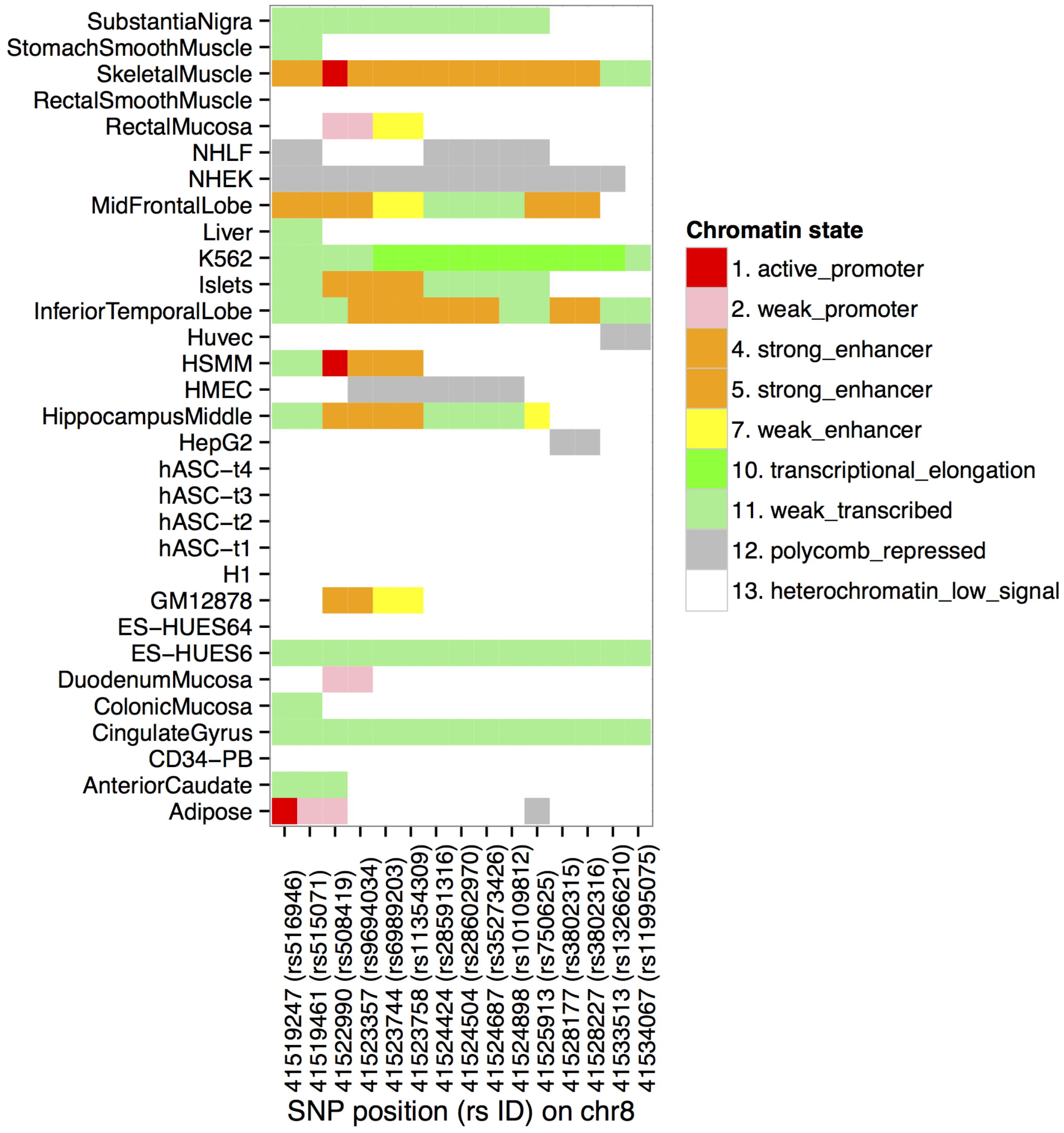

Supplementary Figure 15. Chromatin states overlapping the *ANK1* cis-eQTL T2D GWAS tag SNP rs516946 and those in close ($r^2 \geq 0.8$) LD across diverse cells/tissues. Note that rs508419 overlaps an active promoter state in skeletal muscle and HSMM and is flanked by strong enhancer states.

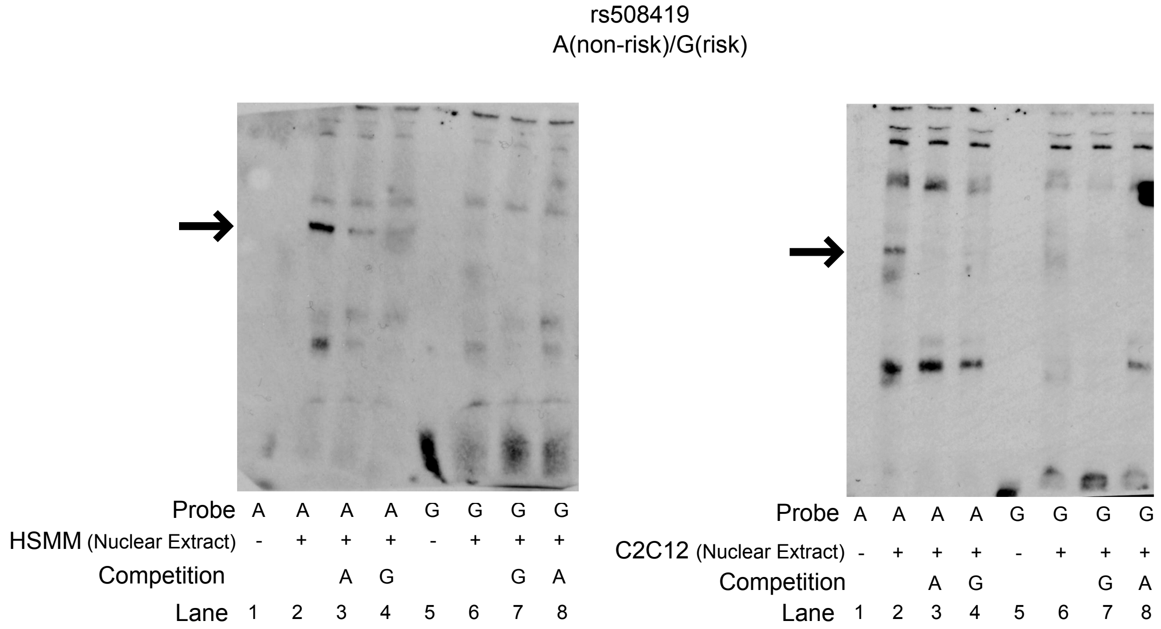

Supplementary Figure 16. EMSA results using human (HSMM; left) and mouse (C2C12; right) myoblast cell nuclear extract.

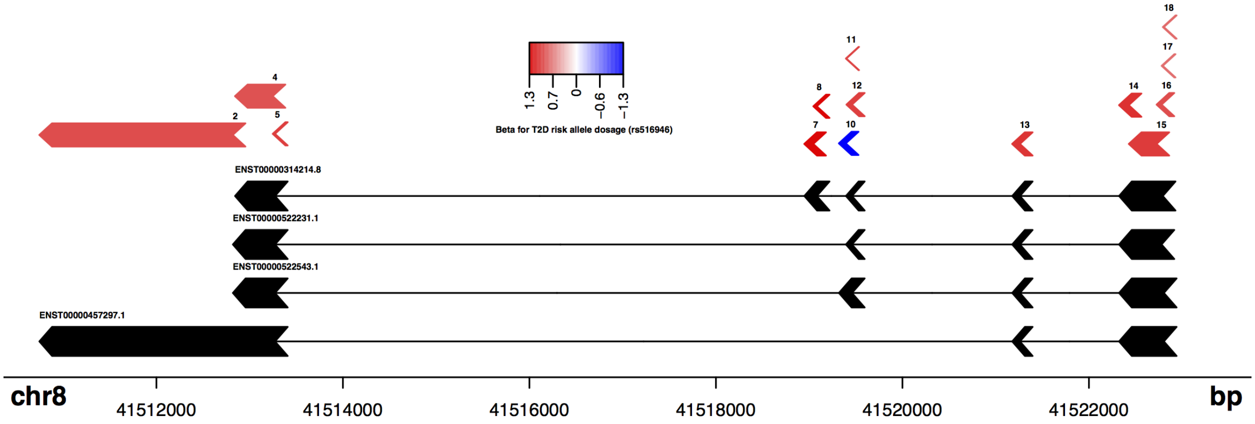

Supplementary Figure 17. We performed an exonQTL analysis on all exonic parts for GENCODE V19 transcripts (see Methods) and show the results for the four short *ANK1* isoforms. Note that exonic part 10 is diagnostic for ENST00000522543.1 and shows decreased splicing with risk allele dosages. These results are consistent sQTL results we report in **Figs. 5bc** and with the RT-qPCR (**Supplementary Figs. 18 and 19**) and ddPCR (**Supplementary Fig. 20**).

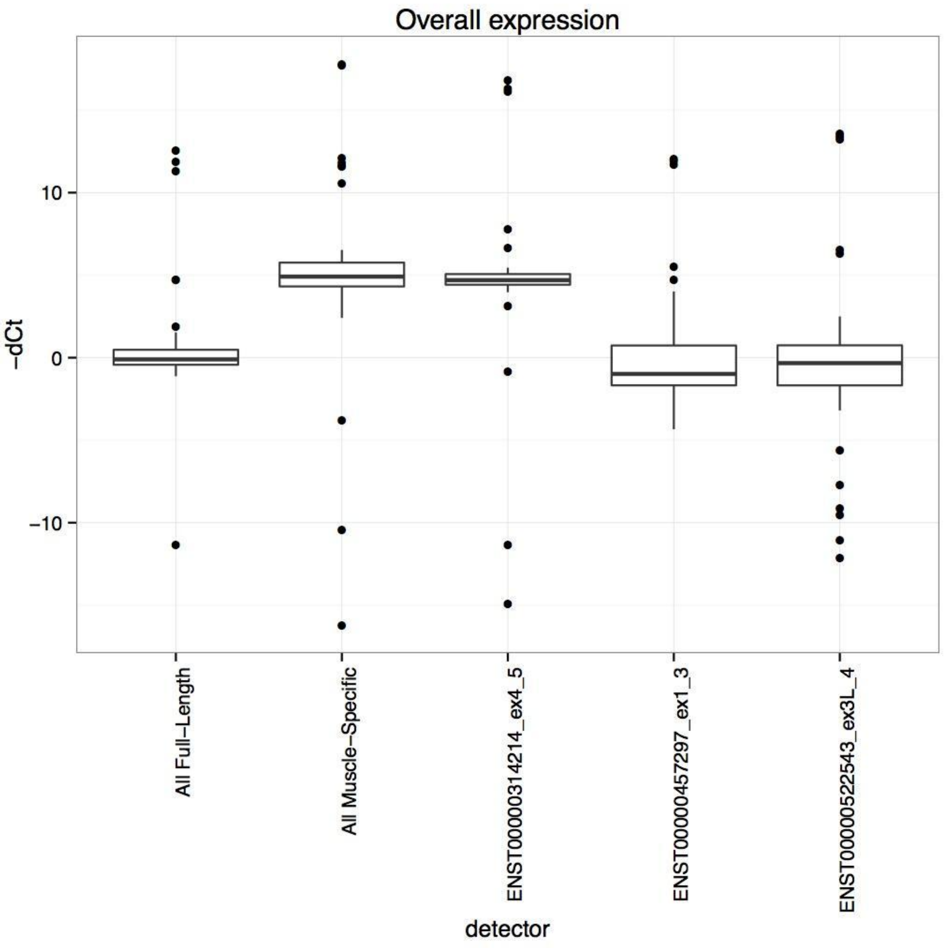

Supplementary Figure 18. RT-qPCR results for *ANK1* isoforms. Twelve samples were assayed, four each from each of the three possible genotype classes at rs508149 (risk allele dosage of 0, 1, or 2). Values are expressed as the inverse of the difference between the Ct values of the target and control (*EMC7*) genes, *i.e.* Ct(*EMC7*) - Ct(target); therefore, greater values indicate higher expression.

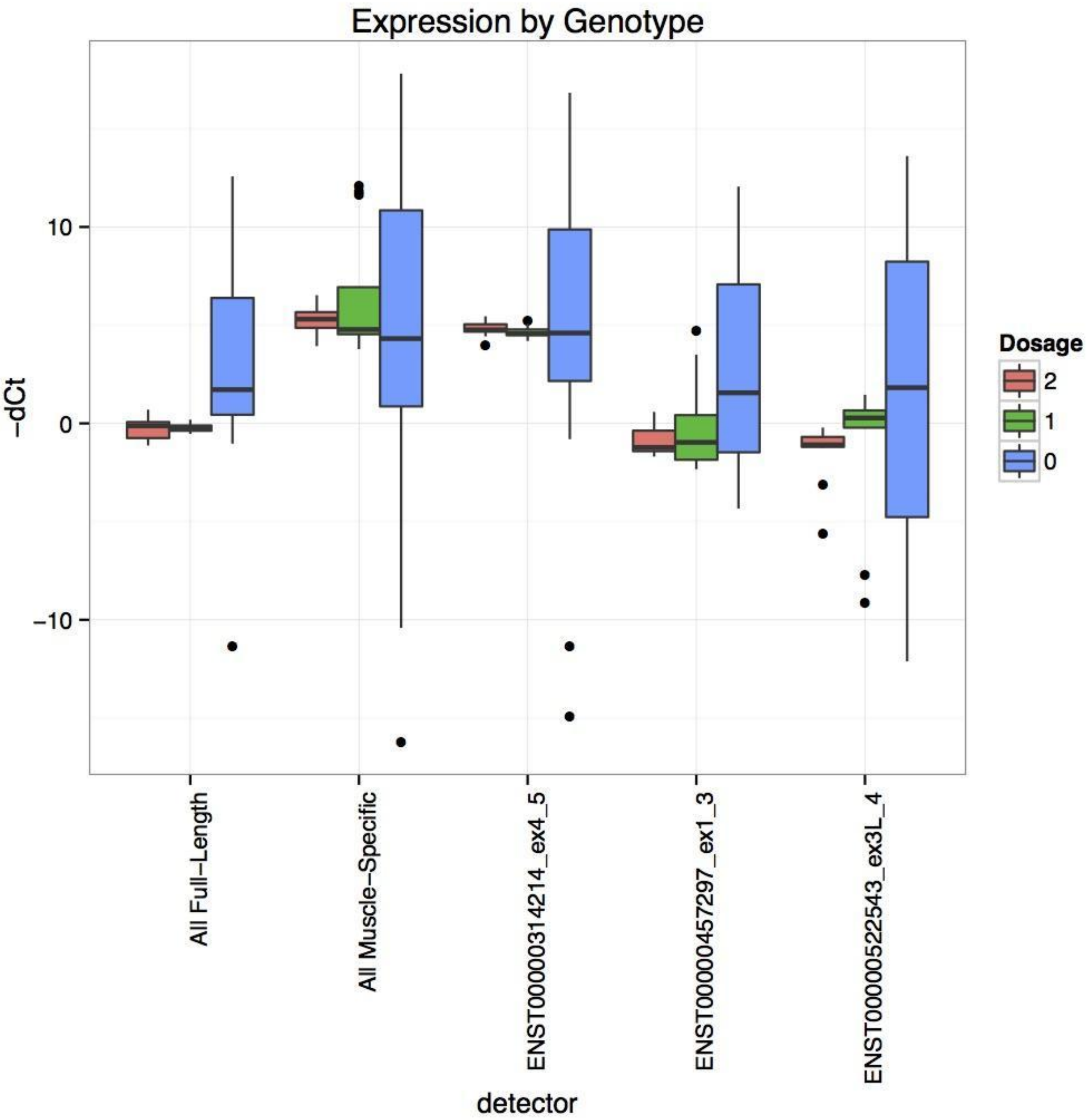

Supplementary Figure 19. RT-qPCR results for *ANK1* isoforms for each of the three possible genotype classes at rs508149 (risk allele dosage of 0, 1, or 2). Values are expressed as the inverse of the difference between the Ct values of the target and control (*EMC7*) genes, *i.e.* Ct(*EMC7*) **-** Ct(target); therefore, greater values indicate higher expression.

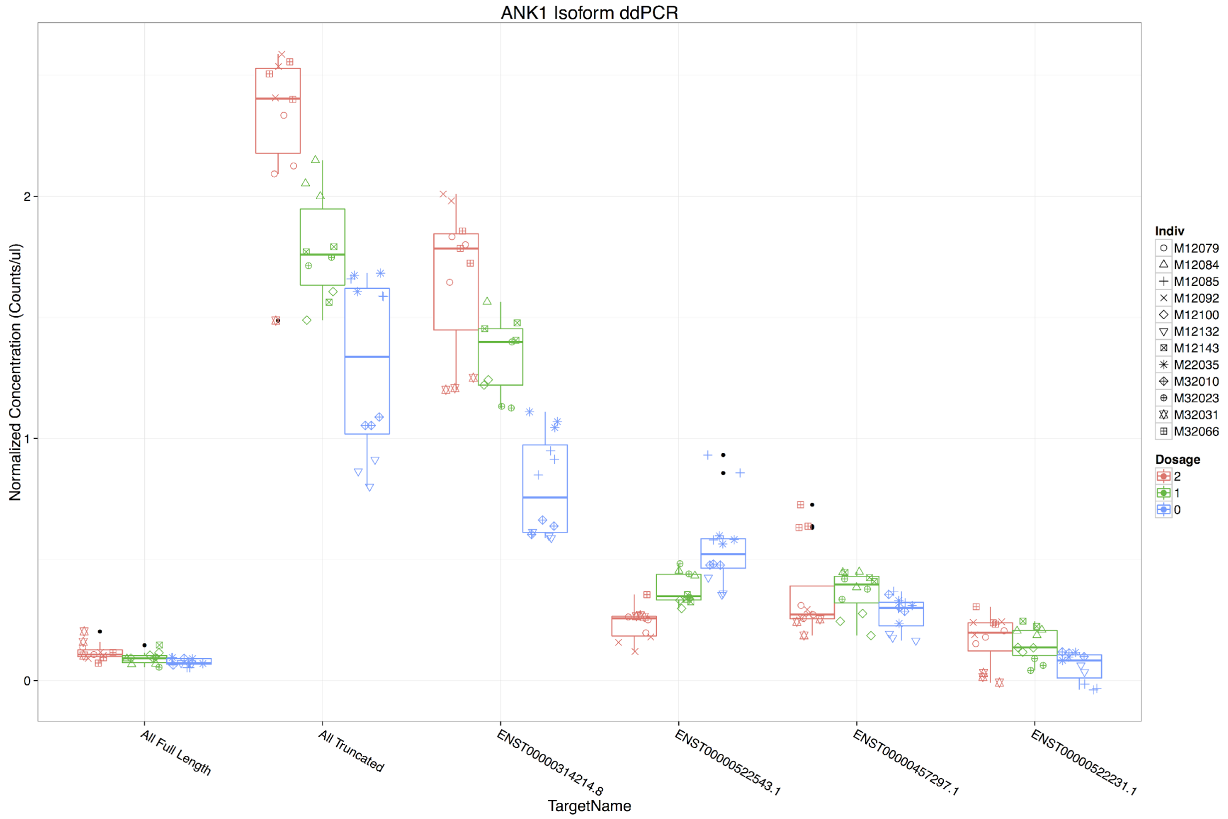

Supplementary figure 20. Results of ddPCR quantification for 12 samples, 4 each with the three possible risk allele dosages. Values are expressed as normalized concentrations and were computed by first subtracting the median of the no-template control and then dividing by the median of the constant assay (*NUCKS1*) within each sample.

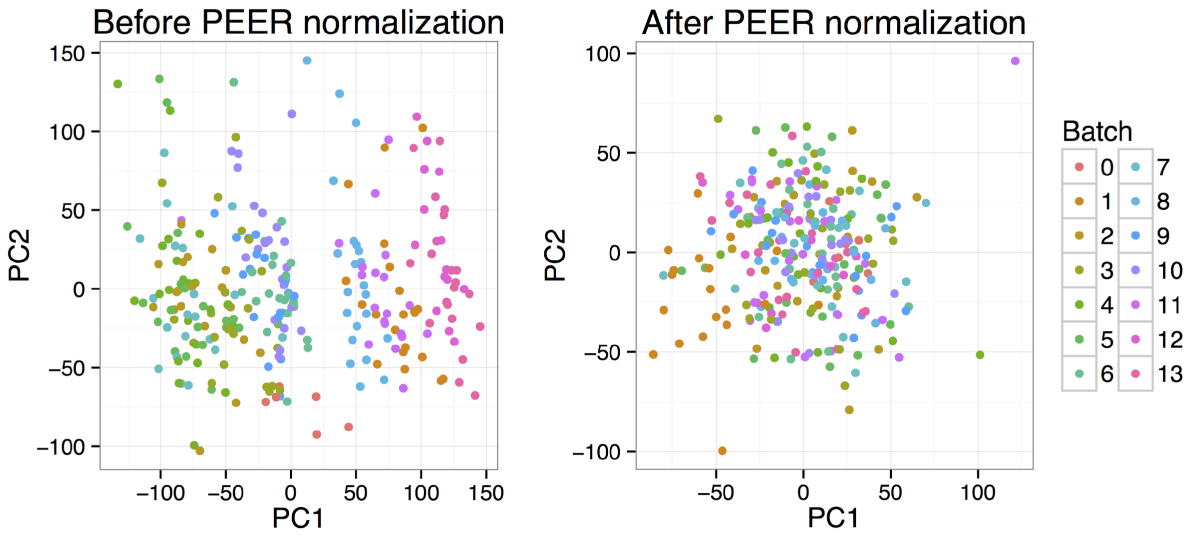

Supplementary Figure 21. Scatter plot of the first 2 principal components on the expression matrix for nuclear genome genes, before (left) and after (right) PEER adjustment. Color-labeling by sequencing batch revealed the presence of systematic batch effects (left). Principal components analysis after normalization using the PEER framework demonstrates removal of batch effects (right).

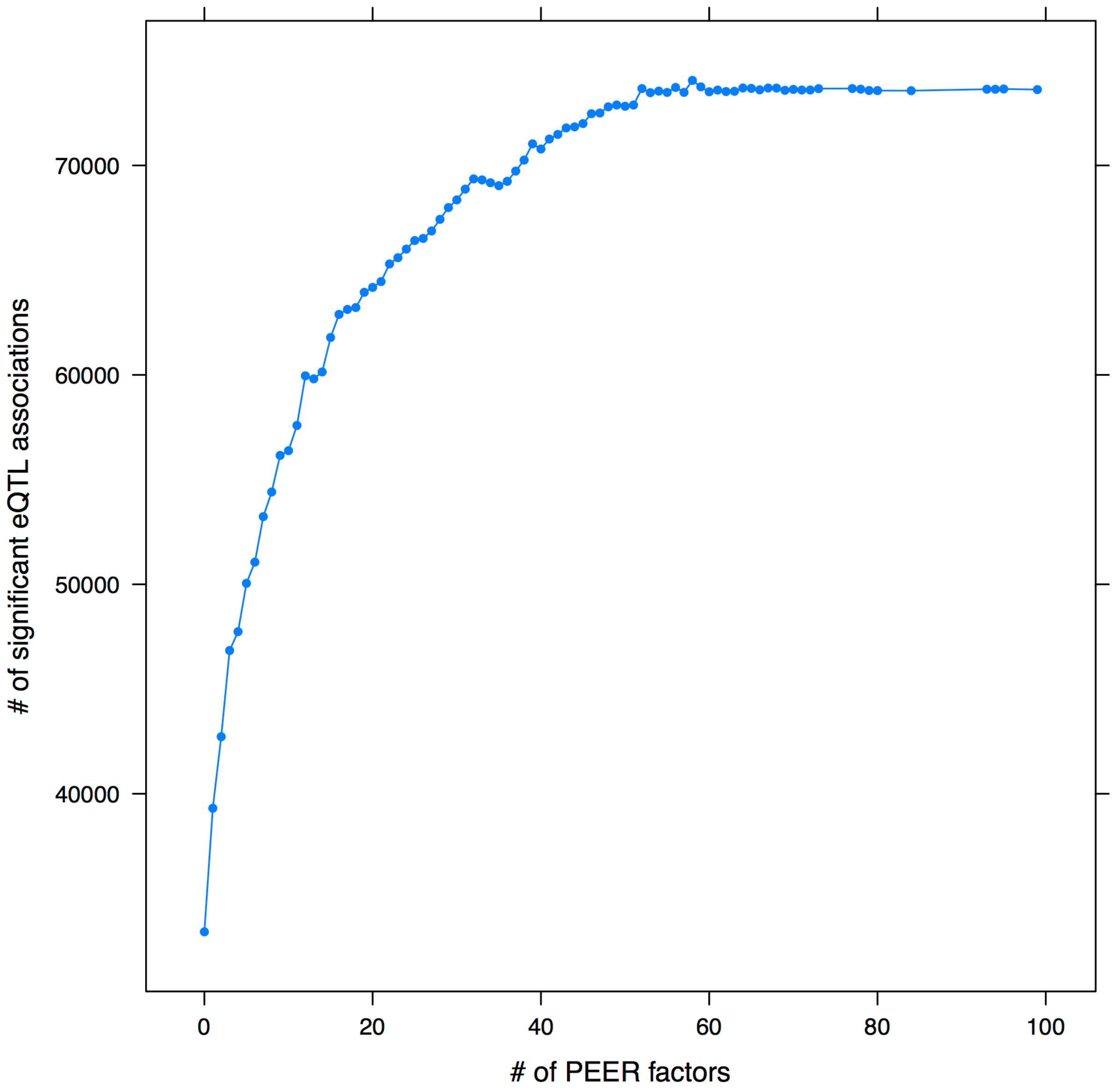

Supplementary Figure 22. cis-eQTL analyses using a diverse number of PEER factors reveals a plateau in the number of significant eQTL associations (FDR=5%) at 60, which is the number we used in our final analysis.

Analysis	Differential Expression					e-QTL
	Total	NGT	IFG	IGT	T2D	Total
N	271	97	36	71	67	267
OGTT Status(n(%))						
NGT	97 (35.8%)	97(100%)	0 (0%)	0 (0%)	0 (0%)	97 (36.3%)
IFG	36 (13.3%)	0 (0%)	36 (100%)	0 (0%)	0 (0%)	35 (13.1%)
IGT	71 (26.2%)	0 (0%)	0 (0%)	71 (100%)	0 (0%)	69 (25.8%)
T2D	67 (24.7%)	0 (0%)	0 (0%)	0 (0%)	67 (100%)	66 (24.7%)
Sex (N, %)	160 M (59.0%) / 111 F (41.0%)	46 M (47.4%) / 51 F (52.6%)	24 M (66.7%) / 12 F (33.3%)	38 M (53.5%) / 33 F (46.5%)	52 M (77.6%) / 15 F (22.4%)	157 M (58.8%) / 110 F (41.2%)
Age (mean(SD)), y	60.1 (7.0)	58.1 (7.0)	57.8 (7.4)	62.9 (5.3)	61.1 (7.3)	60.1 (7.0)
Fasting glucose (mean(SD)), mmol/l	6.26 (0.77)	5.61 (0.33)	6.42 (0.28)	6.19 (0.54)	7.17 (0.65)	6.26 (0.78)
Fasting insulin (mean(SD)), mU/l	8.69 (5.31)	6.72 (3.32)	7.57 (3.46)	10.07 (5.60)	10.67 (6.87)	8.65 (5.27)
WHR (mean(SD))	0.950 (0.077)	0.920 (0.077)	0.945 (0.071)	0.954 (0.073)	0.990 (0.067)	0.949 (0.078)
BMI (mean(SD)), kg/m^2	27.6 (4.2)	26.2 (3.4)	26.4 (3.4)	28.3 (3.8)	29.4 (5.0)	27.6 (4.2)

Supplementary Table 1. Summary statistics for individuals that participated in this study.

	Genes/Exons/Isoforms Tested[b]			Genic Analysis eQTL		Exon Analysis eQTL[d]		Isoform FPKM Analysis eQTL	
**Gene Type Category[a]**	**Genes**	**Exons**	**Isoform FPKM**	**Genes[c]**	**% of Category with eQTL**	**Exons[c]**	**% of Category with eQTL**	**Transcripts[c]**	**% of Category with eQTL**
protein_coding	15,449	353,596	63,158	14,479	93.6	269,871	76.3	34,836	55.2
pseudogene	2,147	14,528	792	1,747	80.5	11,233	77.3	433	54.7
antisense	1,667	7,398	7,597	1,535	92.0	5,878	79.5	3,755	49.4
lincRNA	1,561	6,715	5,536	1,437	91.5	5,330	79.4	2,702	48.8
processed_transcript	244	8,755	845	229	93.9	7,022	80.2	435	51.5
sense_intronic	212	1,319	643	186	87.7	1,037	78.6	320	49.8
sense_overlapping	92	1,252	179	84	91.3	993	79.3	109	60.9
Total	21,420	393,563	78,750	19,697	92.0	301,364	76.6	42,590	54.1

Supplementary Table 2. Count of genes, exons, and isoforms with at least 1 significant eQTL, stratified by gene type. [a]Gene type from GENCODE v19; [b]The total number of gene, exons and isoforms tested for eQTLs (genes with at least 1 tested SNP); [c]Number of genes, exons, or isoforms with ≥1 eQTL; [d]Exons that are annotated to more than one gene are included once in the table for each annotate gene category.
